# Acquisition of temporal order requires an intact CA3 commissural/associational (C/A) feedback system in mice

**DOI:** 10.1038/s42003-019-0494-3

**Published:** 2019-07-03

**Authors:** Brittney M. Cox, Conor D. Cox, Benjamin G. Gunn, Aliza A. Le, Victoria C. Inshishian, Christine M. Gall, Gary Lynch

**Affiliations:** 1 Department of Anatomy and Neurobiology, University of California, Irvine, CA 92697 USA; 2 Department of Neurobiology and Behavior, University of California, Irvine, CA 92697 USA; 3 Department of Psychiatry and Human Behavior, University of California, Irvine, CA 92697 USA

**Keywords:** Hippocampus, Neural encoding

## Abstract

Episodic memory, an essential element of orderly thinking, requires the organization of serial events into narratives about the identity of cues along with their locations and temporal order (what, where, and when). The hippocampus plays a central role in the acquisition and retrieval of episodes with two of its subsystems being separately linked to what and where information. The substrates for the third element are poorly understood. Here we report that in hippocampal slices field CA3 maintains self-sustained activity for remarkable periods following a brief input and that this effect is extremely sensitive to minor network perturbations. Using behavioral tests, that do not involve training or explicit rewards, we show that partial silencing of the CA3 commissural/associational network in mice blocks acquisition of temporal order, but not the identity or location, of odors. These results suggest a solution to the question of how hippocampus adds time to episodic memories.

## Abstract (web summary)

Brittney Cox, Conor Cox, Ben Gunn et al. show that brief stimulation of the commissural/associational fibers elicits extended periods of reverberating activity in the CA3 hippocampal region ex vivo. Partial silencing of the CA3 network in vivo inhibits the acquisition of temporal, but not identity or location, information in mice.

## Introduction

Episodic memory^1,2^ enables the encoding of large amounts of semantic (cue identity), spatial, and temporal material (i.e., what, where, and when information) while allowing for rapid search and retrieval of specific contents. Such memories are acquired as part of everyday life without practice or explicit rewards^3^. Recall and integration of stored episodes are essential to many aspects of cognition including inferential thinking; impairments to these processes are shared characteristics of numerous neuropsychiatric disorders^4–6^.

The hippocampus is critical for the acquisition and use of episodes^7–11^. Moreover, studies of rodents and humans suggest that subsystems within hippocampus are differentially linked to encoding two of the three basic episodic elements. Specifically, lateral entorhinal cortex (LEC) appears to convey cue identity to hippocampus via its lateral perforant path (LPP) projection to the dentate gyrus outer molecular layer^12–16^. Spatial cues preferentially use the medial entorhinal cortex (MEC) and its medial perforant path (MPP) projections to the mid-portion of the same dendrites^15,17,18^. These conclusions align with entorhinal cortex afferents: LEC is innervated by the ventral stream of cortical visual processing, which conveys information about cue identity whereas MEC is targeted by the dorsal stream which relays spatial information^14,19–22^.

The manner in which hippocampus adds the critical temporal dimension to an episodic memory is poorly understood. Learning theories generally posit that events closely associated in time become connected in memory^23^, an assumption that accords with the temporal contiguity requirement (‘Hebb Rule’)^24^ for induction of long-term potentiation (LTP). How then are events separated by tens of seconds or longer associated, as occurs in episodic memory? Studies using prior training have identified multiple hippocampal and entorhinal cortical regions in which neuronal firing changes in a manner indicating temporal processing^25–29^. These findings suggest substrates for encoding time but do not address how temporal order is acquired during the first unsupervised sampling of a cue series. The latter process is essential for formation of human episodic memories and is thought to depend on hippocampus^7^. A potential solution to the problem of bridging widely spaced cues postulates involvement of recurrent neuronal networks capable of maintaining reverberating (self-sustained) activity for extended periods^23,30,31^. The extremely dense hippocampal field CA3^32^ commissural/associational (C/A) system could represent such a network^11,23,30,31,33,34^ but experimental support is lacking.

The present studies evaluated contributions of LEC, MEC, and CA3 to the acquisition of three basic elements of episodic memories, with an emphasis on temporal order. Recordings from hippocampal slices were used to test for prolonged reverberating activity in the C/A system. This led to the discovery that brief inputs increase CA3 pyramidal cell (PC) firing for minutes, a period unprecedented for studies of brain networks. Complex systems of this type are prone to catastrophic collapse and this proved to be the case for self-sustained activity in CA3. Novel behavioral protocols were used in conjunction with regional chemogenetic silencing to determine the extent to which specific hippocampal subfields, including CA3, contribute to extraction of what, where, and when information from multiple cues. Results show that silencing MEC blocked learning of spatial location, as anticipated^15,17,18,35,36^, but without affecting encoding cue identity or temporal order. Silencing LEC prevented acquisition of what information but unexpectedly also blocked retention of when and where; this novel result constitutes evidence that cue identity is fundamental to all aspects of episodic processing. Finally, suppression of the CA3 C/A system blocked acquisition of cue order, leaving cue identity and location memory intact.

Collectively, these results call for a revision to ideas about hippocampal contributions to the basic components of episodic memory and support the hypothesis that reverberating activity bridges the time between events, a fundamental and computationally challenging psychological operation.

## Results

### Olfactory tests for the basic elements of episodic memory

**Fig. 1:**
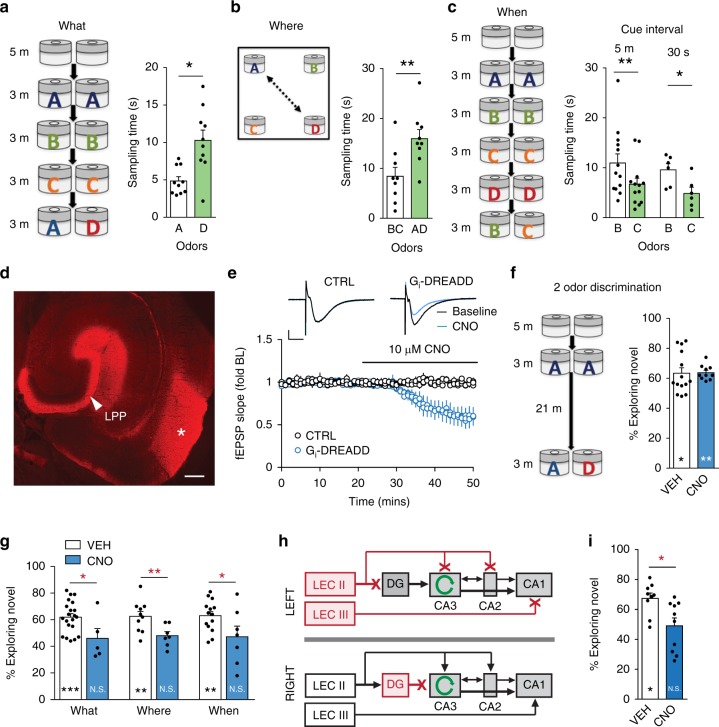
The lateral entorhinal cortex (LEC) is critical for encoding three components of episodic memory. **a** What: The serial odor task tested cue identification: three different odor pairs were sampled in successive 3 min trials spaced by 5 min. At testing mice were exposed to familiar odor A and novel odor D. Mice spent more time investigating the novel odor at testing (*p = 0.016, within group D versus A). **b** Where: Mice sampled 4 odors (5 min) and were tested in a 5 min trial with positions of two odors switched. Mice preferentially explored odors in novel positions (**p = 0.003, within group B,C versus A,D sampling times). **c** When: paired odors were presented in four successive 3 min trials (A:A to D:D) with either 5 min or 30 s between pairs; at testing mice sampled B versus C. Mice spent more time investigating the earlier odor (B) in the series (**p = 0.002, *p = 0.0118, C versus B). **d** Image shows expression of Gi-DREADD associated mCherry in LEC (asterisk) and the lateral perforant path (LPP) projection to the ipsilateral dentate gyrus (bar: 400 μm). **e** Gi-DREADD agonist CNO caused a rapid and marked reduction in LPP synaptic responses; the agonist had no effect in control (CTRL) slices (scale bars: *y* = 1 mV, *x* = 5 ms). **f** Mice were presented with a pair of identical odors and 21 min later with one of these versus a novel odor: Vehicle (VEH)- and CNO-treated mice both spent more time with the novel cue (*p = 0.023,**p = 0.005, within group novel versus familiar odor). **g** Treatment of LEC-DREADD mice with CNO, but not vehicle, suppressed learning in What (VEH: *n* = 21; CNO: *n* = 5), Where (*n* = 15, 7) and When (*n* = 14, 7) tasks (within group comparisons: **p ≤ 0.01, ***p = 0.0009; N.S: *p* > 0.05; between groups: *p < 0.05, **p < 0.01). **h** Design for contralateral disconnect experiments: LEC was transfected on one side and the dentate gyrus (DG) on the other: this leaves unilateral LEC projections to pyramidal cells intact. **i** In the What task, contralateral LPP disconnect mice did not prefer the novel odor when given CNO (N.S. *p* > 0.05) but did so when given vehicle (*p < 0.05; between groups: *p = 0.0119)

Behavioral tests were devised to identify hippocampal regions critical for the acquisition of information regarding the identity, location, and presentation order for olfactory cues. The assays relied on the rodents’ native tendency to investigate novel elements more than familiar ones, thus obviating the need for practice or rewards. The test for acquisition of cue identity (What) involved presenting a sequence of three different odor pairs (A:A, B:B, and C:C) in 3-min sampling sessions spaced by 5 min, followed by a 3-min retention test during which a novel cue was paired with a previously sampled one (A:D). Mice sampled the novel odor more in the retention trial (*p* = 0.02, paired *t*-test; Supplemental Table 1 for behavioral statistics) (Fig. 1a). To test spatial encoding (Where), different odors were placed in each chamber corner; mice sampled the odors for 5 min and were tested 5 min later with positions of two odors reversed. Mice preferentially explored the cues in novel locations (*p* = 0.003) (Fig. 1b). To assess acquisition of the temporal order of cue presentation, mice were given a sequence of four odor pairs (A:A to D:D); at testing they were presented with two of the previous odors (B:C). Mice preferentially explored the cue presented earlier in the sequence (*p* = 0.002) (Fig. 1c). Humans acquire episodic sequences when cues occur in rapid succession or are separated by minutes, indicating that the encoding system possesses an intriguing temporal elasticity^7,15^. Consistent with this, mice performed well in the B:C When test with 30 s rather than 5 min between-cue intervals during sampling of the A–D series (Fig. 1c). Finally, they spent 68.7 ± 6.2% of their time in an A:B retention test exploring odor A (*p* = 0.016 versus B), indicating that cue order was encoded for the full sequence. Supplemental Fig. 1 provides further variations in these paradigms.

### Inhibiting LEC disrupts three aspects of episodic memory

The inhibitory, Gi-coupled designer receptors exclusively activated by designer drugs (DREADD) strategy^37^ was used to transiently inactivate the LEC-LPP system. An AAV construct mediating expression of the hM4Di-DREADD and fluorescent marker mCherry was injected into LEC bilaterally. Verification of injection placement demonstrated dense fluorescent labeling of LEC neurons and their LPP projections (Fig. 1d). We previously showed that administering DREADD agonist clozapine-N-oxide (CNO) blocks acquisition of cue identity in the What task in LEC-DREADD-transfected mice without influencing two-odor discrimination^36^. We repeated those studies and extended the behavioral analysis to evaluate effects on where and when encoding.

Field recordings showed that the size and shape of monosynaptic LPP responses in hippocampal slices from LEC-DREADD mice were comparable to those from naïve controls, but CNO infusion rapidly reduced LPP fEPSP amplitude in slices from transfected mice only (Fig. 1e). In a two-odor discrimination that employed the same postsampling delay as the serial/What paradigm, CNO treatment, and thus bilateral LEC inhibition, had no effect on identification of the novel cue (Fig. 1f), indicating that CNO did not interfere with the animal’s ability to detect or remember odors in a simple discrimination paradigm. In contrast, in LEC-DREADD mice CNO fully blocked learning in the multiple odor What, When, and Where paradigms. For each, vehicle-treated mice strongly preferred the predicted cue and CNO-treated mice did not, and the absolute time spent exploring the predicted cue was different between CNO- and vehicle-treatment groups (Fig. 1g). These effects were only obtained with bilateral Gi-DREADD expression in LEC: With unilateral or missed DREADD injections, CNO had no effect on learning what, where, or when information (Supplemental Fig. 2a–c).

In LEC silencing experiments, the total time spent investigating the odors during initial sampling phases of the What, Where, and When tasks were similar between vehicle- and CNO-treated mice (*p* > 0.05, all comparisons). There was also no effect on distance traveled during spatial task testing (*p* > 0.05) (Supplemental Fig. 2d, e).

Finally, we used a contralateral disconnect paradigm^36^ (Fig. 1h) to test if direct projections from LEC to pyramidal fields of hippocampus proper are sufficient for acquisition in the above behaviors. Specifically, the LEC was silenced on one side and the dentate gyrus on the other, leaving the latter hemisphere with an intact projection from entorhinal cortex to CA3/CA2 (from LEC Layer II) and CA1 (Layer III). CNO treatment reduced behavioral performance in contralateral disconnect mice to chance levels (Fig. 1i) (as noted, CNO treatment of unilateral LEC-DREADD mice did not affect acquisition).

These results establish that information conveyed to hippocampus via the LEC-dentate gyrus projection is critical for acquisition of cue identity, and this is necessary for all three episodic elements.

### MEC selectively supports acquisition of spatial information

**Fig. 2:**
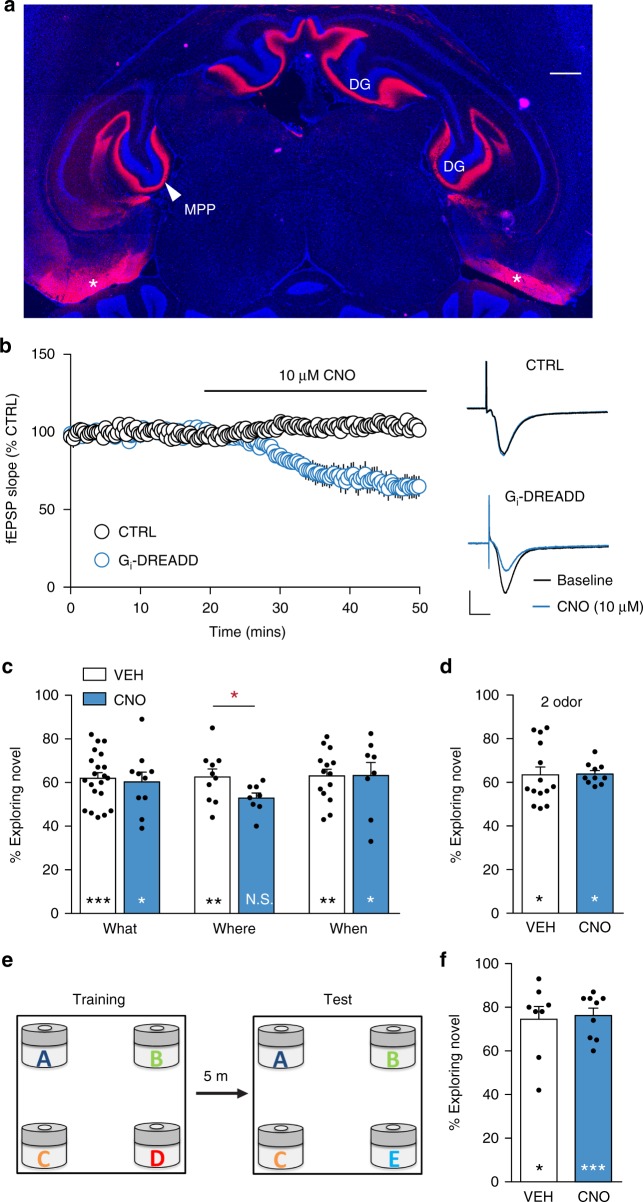
Medial entorhinal cortex (MEC) is selectively involved in encoding where information. **a** A horizontal tissue section shows bilateral expression of Gi-DREADD associated mCherry in the MEC (asterisks) and its medial perforant path (MPP) projection to the dentate gyrus (DG) molecular layer (bar = 400 μm). **b** Plot of MPP fEPSP slopes shows that CNO infusion caused a rapid reduction in MPP synaptic responses in slices from MEC-DREADD mice but not in slices from naïve control (CTRL) mice (mean ± SEM). Representative MPP fEPSP traces recorded in the absence (black) and presence (blue) of CNO in slices from control and Gi-DREADD mice. Scale bars: *y* = 1 mV, *x* = 5 ms. **c** Treatment of MEC-DREADD mice with CNO but not vehicle (VEH) prior to training significantly blocked Where task learning without influencing performance in What or When tests (within group comparisons: *p < 0.05; **p < 0.01; ***p = 0.0009; N.S. *p* > 0.05, paired *t*-tests; CNO groups: What *n* = 7; Where *n* = 8; When *n* = 8; between group VEH versus CNO comparison *p < 0.05, unpaired *t*-test). **d** Transfected mice treated with VEH or CNO both spent more time exploring the novel than the familiar cue in the 2-odor task (*p = 0.022 for VEH, *p = 0.022 for CNO, paired *t*-test). **e** Protocol for testing what memory in the context used to assess where acquisition. **f** MEC-DREADD animals given CNO treatment prior to odor exposure showed a marked preference for a novel cue (what) in subsequent retention tests (VEH *n* = 8, CNO *n* = 9, *p = 0.015, ***p = 0.0006, paired *t*-tests)

Considerable evidence indicates the MEC is critical for encoding spatial relationships between objects^14,15^. We used Gi-DREADD-mediated inhibition to test if MEC is similarly involved in processing information about the locations of multiple odors. Bilaterally transfecting MEC resulted in neuronal labeling and transport of the Gi-DREADD construct to the dentate gyrus molecular layer throughout the septo-temporal extent of hippocampus (Fig. 2a). The efficacy of the Gi-DREADD for suppressing MEC function was verified using field recordings from hippocampal slices. DREADD expression alone did not influence single MPP fEPSPs but CNO infusion rapidly reduced the amplitude of the postsynaptic responses. CNO did not dampen MPP responses in control slices (Fig. 2b).

Behavioral analysis demonstrated discrete effects of suppressing MEC function. In bilateral MEC-DREADD mice, CNO treatment before exposure to the odor cues blocked retention (i.e., blocked preferential exploration of novel location cues) in the Where task (Fig. 2c). Retention scores in CNO-treated MEC-DREADD mice were similar to those receiving vehicle in the What and When paradigms (Fig. 2c) and in the 2-odor discrimination (Fig. 2d). To further test the independence of what encoding from MEC function, we tested acquisition of cue identity using the same 4-cue design used to assess spatial encoding except with one novel odor during testing (Fig. 2e); bilateral MEC silencing had no effect on retention scores (Fig. 2f). As in LEC transfection experiments, vehicle and CNO groups were similar in total times sampling odors during training and test trials (*p* > 0.05, all comparisons). CNO had no effect on distance traveled during training or testing in the Where task (*p* > 0.05, versus VEH) (Supplemental Fig. 2d, e).

These results accord with the consensus view that MEC is required for encoding object location and is not essential for other tasks involving multiple odor cues.

### CA3 C/A system is critical for acquisition of temporal order

We tested the following hypotheses. First, that field CA3, because of its massive collateral feedback projections^32^, operates as a regenerative network capable of prolonged, reverberating activity; and second, that partial silencing of this network will block the encoding of temporal order.

### Reverberating activity in the C/A system

**Fig. 3:**
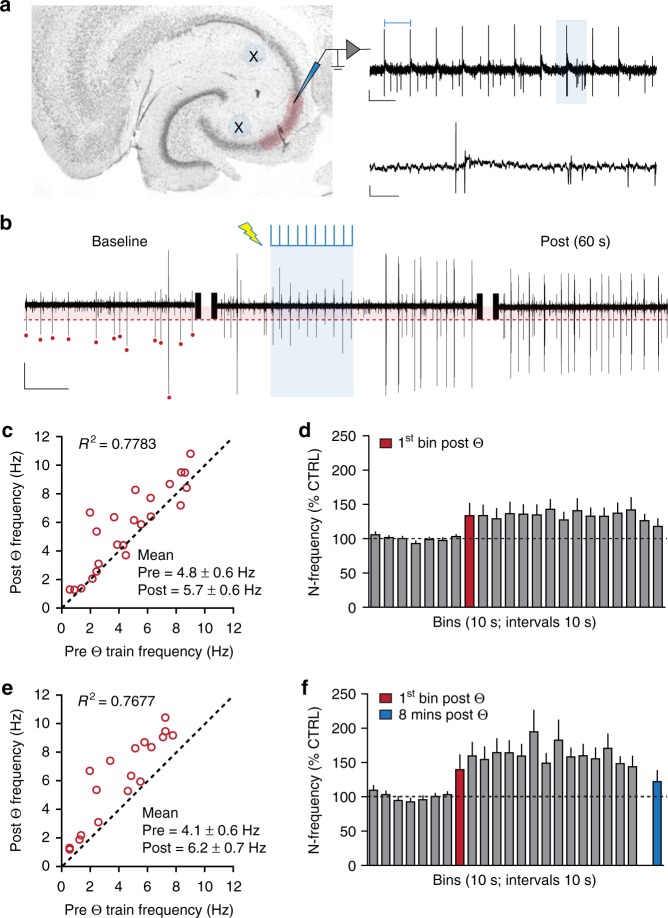
Brief physiological activation of the CA3 C/A system initiates autonomous cell firing lasting minutes. **a** Nissl stained cross section of temporal hippocampus illustrates placement of stimulating (X) and recording (shaded) electrodes. A representative response (right) to a 10-pulse Θ train with stimulation artifacts (top left bar), where an individual fEPSP (from shaded zone) is shown on an expanded time scale below (Scale bars: *y* = 100 µV, *x* = 200 ms and 20 ms for top and bottom traces, respectively). **b** Representative sections of filtered (band pass 300–3000 Hz) extracellular recordings of pyramidal cell spiking during the baseline period (4 s), the Θ stimulation train (shaded blue, 2 s) and subsequent 4 s, as well as 1 min afterward (4 s). The detection threshold and those spikes counted during baseline are indicated with red shading and red dots, respectively. Note that firing frequency is increased following stimulation and this is maintained for ≥ 1 min (Scale bars: *y* = 100 µV, *x* = 1 s). **c** Scatter plot with unity line (dashed) illustrates the mean frequency before and 2 min after Θ stimulation for all cases (*n* = 23 slices; 57 trials). Mean frequency prestimulation and 2 min poststimulation were summarized for all cases (**p < 0.003, paired *t*-test pre versus post). **d** Bar graph summarizing the mean normalized (N) frequency (% control, CTRL) for each 10 s bin during the baseline period and for 4 min after Θ stimulation for 23 slices: group effects for bins were significant (*p* < 0.0001, prestimulation versus poststimulation). **e** Scatter plot with unity line (dashed) summarizes the mean frequency before and for the 2 min after Θ stimulation for positive cases only (*n* = 19 slices; 28 trials). Mean frequency pre- and 2 min post Θ stimulation were summarized for positive cases (***p < 0.0001 prestimulation versus poststimulation). **f** Bar graph summarizing the mean normalized frequency (% CTRL) for each 10 s bin during the baseline period and for the 4 min after Θ stimulation for positive cases (*p* < 0.0001 pre- versus post-stimulation). The blue bar describes mean frequency 8 min after the theta train

Reverberation implies that activating a recurrent network will increase mean firing rates, by any given subpopulation of neurons, for an extended period after terminating the initiating input. We tested for such effects in hippocampal slices, preparations which bypass confounding effects of behaviorally driven stimulation. As expected for a network with dense recurrent connections, there was considerable spontaneous, arrhythmic spiking by CA3 pyramidal cells (PCs), which is absent in CA1 or among dentate gyrus granule cells in hippocampal slices. Two electrodes were positioned to activate separate populations of C/A axons converging on CA3b (Fig. 3a). Following acquisition of stable baseline firing (2.5 min) a brief, physiologically relevant pattern of stimulation (10 pulse Θ-train [5 Hz]) was applied and the subsequent 4 min of PC spiking recorded (Fig. 3b). From 23 slices, a total of 57 trials (i.e., 2–3 Θ-trains separated by 10 min/slice) were analyzed (see *Methods*). The mean spike frequency over the first 2 min post-Θ stimulation was both highly correlated with spike frequency during the 2 min baseline (*R*^2^ = 0.7783) and elevated above the latter (baseline: 4.8 ± 0.6 Hz; post-Θ: 5.7 ± 0.6 Hz; *p* = 0.003) (Fig. 3c. Supplemental Table 2 for physiology statistics). Given the strong pre versus post correlations, we normalized post-stimulation activity to its own baseline to allow for comparisons across trials and slices. Spike frequency was elevated immediately after the short Θ-train and was stable until the last 30 s of the 4-min post-Θ session (Fig. 3d). The strong correlations between prestimulation versus poststimulation periods confirm the stability of the slice preparations and indicate that factors regulating spiking rates continued operating after the short Θ-train.

To better understand the time course for effects of Θ-stimulation, we analyzed spike frequencies for trials in which positive effects (i.e., differences in prespiking versus postspiking) were found. Nineteen (of 23) slices had at least one trial with increased PC spiking frequency after Θ, with positive effects obtained in 28 of the 57 trials. There were no differences in baseline activity between positive and negative trials (positive: 4.1 ± 0.2 Hz; negative: 5.9 ± 0.8 Hz, *p* > 0.05) or in fEPSPs elicited by Θ stimulation. Positive trials were averaged to obtain a single value per slice. As before, the mean spike frequency for the first 2 min post-Θ was both highly correlated with spiking during baseline across the 19 slices (*R*^2^ = 0.7677) and elevated above the latter (baseline: 4.1 ± 0.6 Hz; post-Θ: 6.2 ± 0.7 Hz, *p* < 0.0001) (Fig. 3e). Post-Θ activity increased during the first 30 s to 50–60% above baseline and stabilized for the remainder of the recording session (Fig. 3f). This increase was absent in a 2 min sample (mean of 7 time bins) collected 8 min after the Θ-train (Fig. 3f, *p* > 0.05 versus baseline). There was no reliable increase in PC spiking at any interval following stimulation for negative cases (Supplemental Fig. 3).

Finally, we measured sharp waves (SPWs) to test if prolonged spiking disrupted self-organizing properties of the network. SPW incidence increased by ~20% during the 2 min following Θ stimulation (*p* < 0.015) and there were no changes in mean SPW parameters (Supplemental Fig. 4 and Supplemental Table 3). These results indicate that recurrent activity does not disturb normally occurring network level events.

### Initiation of prolonged CA3 spiking

As described, the brief Θ-train elicited prolonged firing in ~50% of the experiments. We postulated that fluctuations in membrane potentials, due partly to continuous and substantial input from neighboring PCs and stochastic release from the mossy fibers, underlie this variability. Such effects would not be synchronized, resulting in time varying percentages of PCs that are near spike thresholds. Possibly then, Θ-trains applied when a larger fraction of the network is slightly hyperpolarized would be less likely to initiate extended reverberating activity. This argument would require such activity to override the effects of ongoing shifts in membrane potentials.

**Fig. 4:**
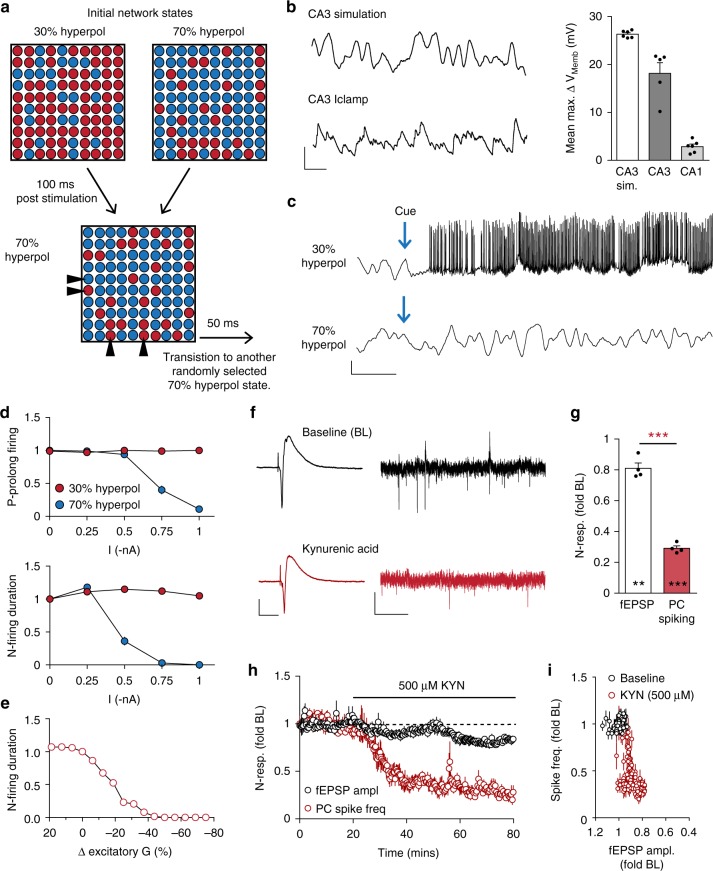
Factors regulating initiation and maintenance of reverberating activity within the CA3 network. **a** Schematic shows initial network states (30 and 70% hyperpolarized) and the distribution of simulated pyramidal cells (PCs) with relatively depolarized (red) or hyperpolarized (blue) membrane potentials. After initial hyperpolarization, 70% of PCs were randomly selected, every 50 ms, to receive 50-ms injections of hyperpolarizing current for the duration of the run. Note, some PCs (triangles) do not change membrane potential with any given transition between states. **b** Exemplar sections (2 s) of simulated (top) and current clamp (Iclamp; bottom) recordings from a CA3 PC illustrating similar amplitude fluctuations in membrane potential during periods without action potentials. Bar graph summarizes the mean (±SEM) maximum change in membrane potential for simulated (*n* = 6), clamp-recorded CA3 (*n* = 6) and CA1 (*n* = 7) PCs. Scale bars: *y* = 10 mV, *x* = 200 ms. **c** Representative sections (4 s) from 4 simulated PCs demonstrate that prolonged firing was only observed when the network was in the 30% hyperpolarized state at time of stimulation (Cue). Scale bars: *y* = 2 mV, *x* = 500 ms. **d** Graphs summarizing how probability (P) of prolonged firing (top) and duration of PC spiking (bottom) are influenced by the magnitude of hyperpolarizing currents in the two initial network states. **e** The hippocampal simulation displays catastrophic collapse following a small (~15–20%) reduction in excitatory transmission in CA3. **f** Representative CA3 fEPSPs (left) and traces of PC spiking (right) in the absence (black) and presence (red) of kynurenic acid (KYN; 500 µM). The sharp negative deflection is the antidromic potential. Scale bars: fEPSP y = 1 mV, x = 10 ms; PC spiking y = 100μV, x = 200 ms. **g** Graph summarizing the unequal effects of KYN upon fEPSP amplitude and PC spike frequency (*n* = 4): **p = 0.0014 for effect on fEPSPs; ***p = 0.0002 for effect on PC spiking; ***p = 0.0007 for difference in KYN effect on fEPSP versus spiking. **h** Time course of KYN effects upon fEPSP amplitude and PC spiking. **i** Graph of the reduction of PC firing versus the decrease in fEPSP size during KYN infusion illustrates the catastrophic collapse in CA3 activity; as in the simulation, a <20% reduction in excitatory transmission caused a drastic reduction in PC spiking (*n* = 4)

We evaluated this proposal by modifying a previously described CA3 simulation, composed of 1000 Pinsky–Rinzel neurons^38,39^, which generates SPWs. The modifications increased local connectivity to generate recurrent firing. Hyperpolarizing 1 nA current injections were delivered to a random 30% or 70% subpopulation of simulated PCs to generate each of the two initiation states (Fig. 4a, top). Following initial activation (100 ms) and for the remainder of the run, subpopulations of PCs (70% of total) were randomly selected every 50 ms to receive injection of hyperpolarizing current (Fig. 4a, bottom). Such current injections produced membrane voltage shifts comparable to the large fluctuations observed during current clamp recording of CA3 but not CA1 PCs (Fig. 4b). To mimic extracellular recording, we sampled membrane voltage from a local group of four neurons in the CA3 model. Activation failed to produce repetitive spiking when the majority of neurons in the network were in the 70% hyperpolarized state but caused seconds long increases in firing when the system was in its 30% condition. Elevated PC firing persisted despite the network transitioning to and remaining in the 70% hyperpolarized state (Fig. 4c). Tests for the magnitude of current injections needed to produce these effects indicated that 0.75 nA, when present in 70% of cells during afferent stimulation (i.e., the 70% hyperpolarized state), reduced the likelihood of generating prolonged activity by ~50% (Fig. 4d top, blue); 0.50 nA shortened the persistence of firing (Fig. 4d bottom, blue). There was no evident relationship between current injection size and repetitive activity when stimulation was delivered in the 30% hyperpolarized state (Fig. 4d, red).

Complex systems with positive feedback are prone to catastrophic collapse or runaway activity with small changes in connection strength. Accordingly, a simulated 20% reduction in conductance of excitatory synapses nearly eliminated the prolonged firing (Fig. 4e). We tested the point in hippocampal slices, using ionotropic glutamate receptor antagonist kynurenic acid (500 µM) which reduced C/A elicited fEPSPs and PC spiking by ~20% and >70%, respectively (Fig. 4f, g). A plot of the two measures over time emphasizes the extent to which firing frequency collapses with relatively small changes in transmission (Fig. 4h, i).

### Unilateral CA3 silencing and temporal order acquisition

**Fig. 5:**
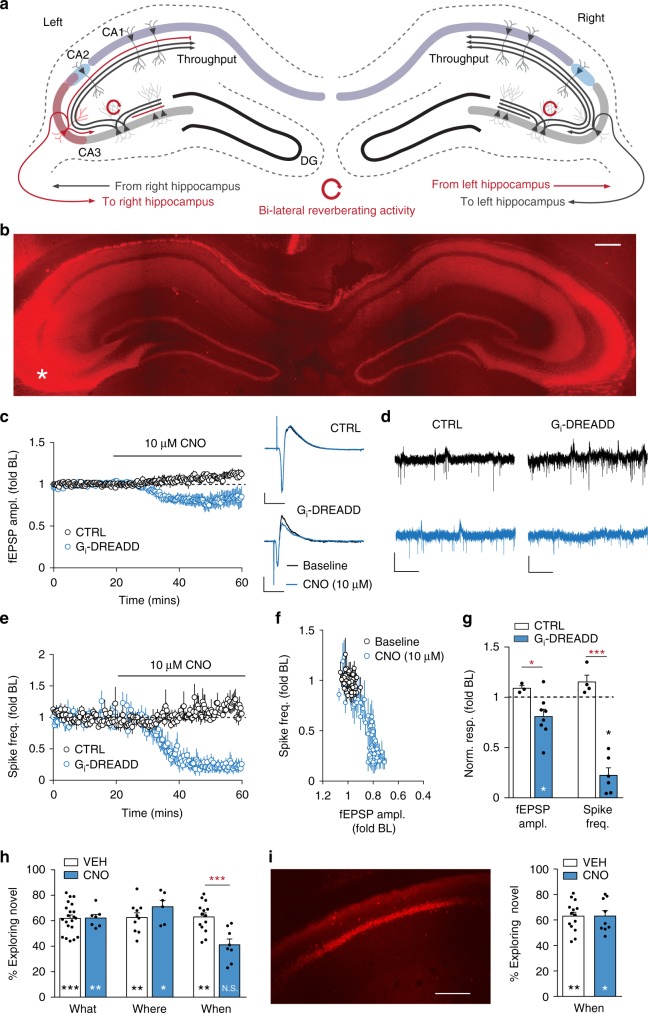
Unilateral silencing of CA3 neurons selectively disrupts acquisition of temporal information. **a** Schematic of experimental design. CA3 receives afferents from entorhinal cortex and dentate gyrus, and monosynaptically innervates CA2 and CA1. This constitutes a rapid and potent throughput network. CA3 collaterals form a dense ipsilateral, associational network capable of prolonged self-sustained activity. The two sides are linked by massive commissural projections that provide ~50% of pyramidal cell (PC) input to CA3 strata oriens and radiatum. Ipsilateral silencing of a portion of CA3 thus removes a significant portion of the feedback system bilaterally (red lines), a condition shown to cause catastrophic collapse of reverberation (red circles). **b** Image shows bilateral hippocampal distribution of mCherry after unilateral AAV-Gi-DREADD-mCherry transfection of CA3a,b (asterisk marks injection placement; bar = 400 μm). **c** Plot of Schaffer-commissural fEPSPs shows CNO modestly reduces fEPSP amplitude (ampl) in transfected slices without effect in non-transfected controls (CTRL). Representative traces at right (Scale bars: *y* = 1 mV; *x* = 10 ms). **d** CA3 PC spiking before (black) and 40 min after (blue) CNO infusion in slices from control versus Gi-DREADD hippocampi. Scale bars: *y* = 100 µV, *x* = 200 ms. **e** Time course for CNO effects on mean spike frequency (±SEM) in slices from control versus Gi-DREADD mice. **f** Graph shows CNO causes catastrophic collapse in CA3 activity in slices from CA3-DREADD mice (*n* = 6, *p* = 0.0003 fEPSP versus spiking). **g** Effects of 40-min CNO infusion on fEPSP amplitude (CTRL: *n* = 3, DREADD: *n* = 8) and spiking (CTRL: *n* = 3, DREADD: *n* = 6) (*p ≤ 0.05 paired *t*-test, baseline versus CNO. Between group comparisons: *p < 0.05, ***p < 0.0001, unpaired *t*-test, CTRL versus DREADD). **h** CA3-DREADD mice given CNO before odor sampling did not show the normal preference for earlier versus later odors during When retention testing (*n* = 8, N.S. *p* > 0.05, paired *t*-test); Vehicle (VEH) injections had no effect (**p = 0.01). VEH versus CNO: ***p < 0.001 for time sampling earlier odor (unpaired t-test). CNO did not affect What (*n* = 7) or Where (*n* = 6) acquisition in CA3-DREADD mice (within group comparisons, time with novel cues or locations, respectively: *p = 0.02; **p < 0.01, ***p = 0.0009, paired *t*-test). **i** Unilateral Gi-DREADD silencing of CA1 (representative image, bar = 400 μm) did not impair When acquisition (CNO: *n* = 9, *p* > 0.05, paired *t*-test)

Results described above indicate that unilateral operation of the entorhinal–dentate gyrus system is sufficient for mice to acquire what and where elements of an episode. Notably, both LEC and MEC projections are overwhelmingly directed to the ipsilateral hippocampus^16^. However, CA3 receives ~50% of its PC input from the contralateral side via the massive commissural system^34^. The sensitivity of the network to relatively small losses of excitatory transmission strongly suggests that partial silencing of CA3 within one hemisphere will cause a bilateral reduction of recurrent firing. However, rapid throughput across the dentate gyrus-CA3-CA1 circuit would be minimally affected on the non-transfected side (Fig. 5a). Therefore, unilateral suppression of CA3 can be used to evaluate behavioral functions of the C/A network while leaving identifying cues and their locations intact.

Accordingly, CA3a/b was unilaterally transfected with the inhibitory Gi-DREADD construct in dorsal and ventral hippocampus (two injections). This resulted in expression by PCs across much of the septo-temporal extent of CA3a/b and dense transport to ipsilateral and contralateral C/A targets (Fig. 5b). We prepared hippocampal slices from CA3-DREADD mice after behavioral testing and confirmed that CNO rapidly reduced (<20%) the amplitude of fEPSPs, elicited by stimulation of C/A projections, in recordings collected outside the zone containing transfected cell bodies; CNO had no effect on fEPSPs in control slices (Fig. 5c). The magnitude of the CNO effect on synaptic responses reflects the absence of transfection in a substantial percentage of ipsilateral and contralateral CA3 neurons that project to the recording site. In contrast to the modest effect on synaptic responses, CNO caused a profound reduction in cell firing recorded from the same electrode used to sample the positive end of the fEPSP dipole (Fig. 5d, e). We estimated the relationship between the % reductions in fEPSPs and CA3b spiking by measuring the two variables at various intervals after CNO infusion onset (Fig. 5f) and obtained evidence for a catastrophic collapse when fEPSPs were decreased by ~20% (Fig. 5g). We conclude from these analyses that the spatially restricted transfections had a relatively modest effect on throughput across field CA3 but produced a profound reduction in the capacity of the network to generate recurrent activity.

Unilateral CA3-DREADD mice given CNO did not acquire temporal order in the When task. Vehicle-treated mice spent more time investigating the earlier odor (B > C) in the previously sampled cue series (*p* = 0.010), whereas CNO-treated mice did not (*p* > 0.05). The percent time exploring the earlier cue was significantly lower in CNO versus vehicle groups (Fig. 5h When: *p* = 0.0006). In contrast, in mice with unilateral Gi-DREADD transfection of CA1, CNO did not disrupt the ability to detect cue order (Fig. 5i).

For unilateral CA3-DREADD mice, there were no differences between those receiving CNO versus vehicle in time spent investigating odors during training or in exploration of the arena in the Where task (Supplemental Fig. 2d, e). We conclude that partial field CA3 inhibition, and the associated disproportionate depression of cycling activity in the C/A network, selectively interferes with acquisition of temporal order.

## Discussion

Episodic memory involves forming associations between elements in a sequence without prior training or current rewards; it minimally requires semantic, spatial, and temporal information: what, where, and when. Tests of episodic memory in humans have used diverse paradigms ranging from discrete cues to complex stories^7,15,40^. A recent version involved individuals taking a first time, ~25-min walk across a university campus during which several events occurred. Recall of individual items, their locations, and their order of occurrence was assessed directly afterwards. Acquisition of the three types of information was defective in individuals with hippocampal damage but encoding temporal order was particularly impaired^7^. We developed protocols for rodents that incorporate key features of this experiment to identify a hippocampal subsystem related to temporal ordering. Mice were allowed to sample a series of odors, cues of innate interest to rodents, separated by minutes; they were tested for retention of what or when data shortly afterwards. A separate paradigm was used to evaluate unsupervised learning of odor locations. After establishing that control mice perform proficiently without prior training or rewards, we used chemogenetic techniques to determine if particular hippocampal pathways are selectively involved in the different components of an episodic memory.

Bilaterally silencing of the MEC severely impaired spatial learning without affecting what and when acquisition. These results constitute evidence that rodents combine visual-spatial information with cue recognition to learn the relative positions of multiple odors. The findings highlight the animals’ ability to connect what and where (<identity-location>) aspects of environmental inputs. Field CA3 with its dense collateral system is a likely site for convergence. The two types of data occur in close temporal proximity during sampling, which substantially reduces the amount of processing within CA3 needed for combination of signals. This hypothesis is consistent with the proposal that CA3 acts as an auto-associative, pattern completion network for complex cues^41–45^.

Bilateral LEC silencing thoroughly disrupted retention in the serial odor What task without disturbing encoding of a single-odor discrimination. The question thus arises as to why the LEC is needed for performance in serial but not single cue paradigms. Contralateral disconnect experiments showed that projections from LEC to the dentate gyrus are required for the more complex serial task. It therefore appears that animals rely on entorhinal-hippocampal associations, rather than plasticity available in the upstream piriform cortex^46,47^ to meet the greater demands for encoding sequences. A clear adaptive advantage for this switch in processing modes is suggested by results of the MEC experiments: it affords an opportunity for associating cue identity with location.

Transient LEC suppression also blocked performance in Where and When paradigms, a somewhat surprising result, although both tasks require the animal to maintain the identities of multiple recently sampled odors, and the latter process is blocked by silencing the LEC.

We then tested if CA3 is capable of generating responses of sufficient duration to bridge the delays between serial cues, as routinely required by episodic memory^7,48,49^. Prior studies have described neuronal populations that perform timing operations within multiple regions of the entorhinal-hippocampal system^50–56^. Specifically, tasks using rewards and/or extensive training have suggested that cells located in LEC^25^ and CA2^26,27^ impart temporal information associated with an animal’s location. Roles for Layer III MEC neurons in the maintenance of temporal traces^28^ and the emergence of CA1 time cell sequences^50^ have also been described. Our studies tested if the bilateral associational system generated by CA3, a feature lacking in CA2 and CA1^34,57^, produces singular physiological patterns that could acquire temporal order during unsupervised sampling of a sequence of novel cues, issues not addressed in prior work.

The idea that CA3 is critical for encoding the when element of an episode relates to the much discussed hypothesis that large populations of interconnected neurons (cell assemblies^58^) can generate reverberating activity and thereby maintain a temporally extended representation of a brief input. Such systems are thought to provide a mechanism for working memory^45,59–61^. The cycling activity concept was developed from neuroanatomical considerations by Lorente de No^62,63^ and used as a second postulate in Hebb’s influential theory of memory^58^. Reverberation also figured prominently in early neural network models^64^ and has since been a topic of intense computational and theoretical work^61,65–67^. Convincing evidence for self-sustained activity in brain came from unit recordings in primate frontal cortex during performance of a working memory task^59^: firing generally lasted for a few seconds, as expected for working memory, but it is unclear that the effects reflect the type of reverberation envisioned by Hebb and others. Our experiments determined that the unusually dense interconnections within CA3 are capable of maintaining autonomous activity for much longer periods: a 2-s input initiated firing that persisted for minutes. Given that recordings were collected in an isolated preparation without peripheral input and were sensitive to modest changes in the network, it can be concluded the prolonged activity was intrinsically generated.

Post-hoc analyses failed to uncover reasons why prolonged CA3 neuronal firing did not occur in every trial, even within a given slice. But the observed variability accords with the argument that reverberating systems are inherently dynamic with spontaneous transitions between multiple states. Simulations suggested that changes in membrane potentials, normally occurring at random among CA3 neurons, frequently create conditions that are nonconducive to reverberation. The models also satisfied the related expectation that reverberation will be sensitive to changes in connection strength, which was confirmed in physiological experiments. These results suggested a means for avoiding a major difficulty facing attempts to use experimental manipulations to investigate contributions of the CA3 C/A system to memory. Bilateral silencing of a substantial portion of CA3 would be expected to interrupt the primary hippocampal circuit (DG-CA3-CA1) and thus block most processing performed by the structure. In contrast, unilateral silencing of a portion of CA3 would eliminate a substantial proportion of the bilateral C/A system, and thus reduce cycling (reverberation) between hemispheres, while leaving throughput intact on the contralateral side.

Unilateral chemogenetic/DREADD silencing of a portion of CA3 PCs blocked retention of the temporal order in which cues were sampled without disturbing what and where encoding, thereby confirming that opposite-side processing, including integration of cue identity and spatial information, was intact. Note that time-related activities in LEC, CA2, and CA1 would also be operational on the contralateral side indicating that they are insufficient for retention of temporal order. The What and Where paradigm results for mice with unilateral CA3 silencing demonstrate that the animals can remember odors presented in a sequence and their locations despite suppression of the C/A system. These results indicate that bilateral CA3 system is essential for attaching temporal information to the representations of the cues <identity-time>.

In vivo testing for prolonged recurrent activity in the When paradigm will face the problems of establishing precise onset times during unsupervised sampling of odor cues and distinguishing activity due to reverberation from that produced by ongoing behavior. Without chronic recording data, we can minimally conclude that temporal ordering but not identity and location processing requires an intact, CA3 C/A system. Given that limited CA3 silencing profoundly reduced reverberation in slices prepared from the same mice used for behavioral testing, with small changes to throughput, it is reasonable to assume that the essential contribution of CA3 to acquisition of temporal information in the episodic memory tests involves the unusual capability of the subfield to generate self-sustained activity.

It seems unlikely that the recursive CA3 network maintains firing associated with each cue, and keeps these segregated throughout the sampling periods and delays before retention testing. A more plausible explanation is the prolonged spiking produces a transient potentiation of synapses between CA3 neurons participating in the odor-initiated cycling, and this effect could persist for the duration of the sessions. Possibly related to this is evidence that prolonged firing is associated with an increased incidence of SPWs, large depolarizations hypothesized to promote synaptic modifications^68^. The steady decay of cue-induced synaptic response enhancement would be proportional to the position of the cue in a sequence, thereby providing a relative novelty basis for distinguishing between cues in the retention trial. An alternative hypothesis for order encoding is that prolonged CA3 firing could link populations of neurons activated by successively presented cues. In this scenario, presenting the two odors together in the retention test would result in a stronger response to the later cue in the original sequence and thus greater perceived novelty of the earlier cue. The decaying traces hypothesis predicts that retention scores (B versus C) will improve with longer between-cue intervals during initial sampling—the trace for B will have decayed further before C appears. However, reducing the delay between odors from 5 min to 30 s during sampling did not impair task performance. Moreover, the mice readily distinguished odors A versus B in the retention trial, a comparison that occurred when the relative difference in decaying memories would be minimized. Independent of mechanisms, this result demonstrates that mice acquired information about the sequence in which A:B:C had previously occurred, thereby providing a substrate for the temporal ordering required for episodic memory. Finally, decaying trace and cue linkage hypotheses are not mutually exclusive: relative contributions of the two processes during retention testing could reflect the intervals separating cues during prior learning.

Information assembled within CA3 is transferred to CA2 and CA1, and CA1 directly and indirectly innervates MEC/LEC^16^. It is difficult to assess the likelihood that the CA3 effects described here interact with timing capabilities described for each of these downstream network nodes^26,27,50^ because of marked differences in the behavioral paradigms used in past versus present studies. It is however reasonable to assume that the within-session, experience-related firing reported for LEC^25^ shapes CA3 reverberation, which in turn, when filtered by CA2/CA1, promotes temporally extended LEC activity. Similarly, CA2 activity following cue sampling, as described in unit recording experiments^26^, would promote bilateral reverberation in CA3 initiated by that cue and transferal to CA1.

With regard to the above, our results indicate that mice link cue identity with spatial and temporal information, two operations that could potentially occur in CA3 as a type of pattern formation, with the resultant cue-location or cue-time signals relayed to CA1. The latter region constitutes a logical site for integrating these inputs into a minimal what, where, and when episodic experience. But performance in each of three tasks developed for this project may also require generation of a second type of data: relative novelty of cues. CA1 is a likely site for this function as numerous studies have firmly associated this subfield with novelty detection^69,70^. We accordingly propose that hippocampus is engaged as animals are confronted with a simple sequence of cues during unsupervised exploration (What) and employs additional subdivisions as complexity increases. The MEC provides information required for placing multiple elements in space whereas the singular CA3 C/A network preserves the sequence, in both cases without cue identity loss. Importantly, the proposal posits that rodents use perceived novelty to register cue order rather than to recall entire sequences. We suggest that the hippocampal mechanisms described here constitute substrates that enable the more elaborate processing of episodic experience found in humans.

## Methods

### Animals

Studies used 2–6 months old male mice (FVB-129 and C57BL6 backgrounds) group housed (3–5 per cage) with food and water ad libitum. Mice were on a 12 h on/12 h off light cycle with lights on at 6:30 AM. Experiments were conducted in accord with NIH guidelines for the Care and Use of Laboratory Animals and protocols approved by the Institutional Animal Care and Use Committee.

### Behavioral tests

Paradigms used olfactory cues to evaluate the what, where, and when components of episodic-type memories in FVB-129 mice.

*What*: A serial odor task was used to assess encoding of cue identify (what) information (see Fig. 1a). Odorants were diluted in mineral oil and 100 µl of the scented mixture was pipetted onto filter paper (final concentration of 0.1 Pascals) which was placed in a glass jar (5.25 cm diameter × 5 cm height) with a plastic lid containing a ~1.5 cm diameter hole to allow the mouse to explore the odorant. During the habituation session, the mouse was allowed to explore for 5 min a plexiglass arena (30 × 25 cm floor, 21.5 cm walls) containing two cups (without odor). The mouse was then moved to an identical empty chamber for 5 min while the cups in the test arena were replaced with cups containing odor A (in duplicate) for the next session. After 3 min exploration, the mouse was again moved to the holding chamber for 5 min and the cups with odor A in the test chamber were replaced with cups containing odor B. This sequence was repeated with odor C. In the final test session 5 min later the mouse was exposed to two different odors: a previously sampled odor A and a novel odor D. The time spent sampling D versus A was used as a measure of retention.

*Where*: This paradigm used a large plexiglass arena (60 cm × 60 cm floor, 30 cm walls) containing four odor cups (see Fig. 1b). Mice were habituated to the chamber for 5 min with cups not containing odors and then transferred to an alternate identical empty holding chamber for 5 min. During the following training session the mouse was returned to the initial arena and allowed to explore cups scented with four different odors for 5 min. After placement in the holding chamber (5 min) the mouse was returned to the original arena in which the location of two of the four scented cups had been switched. In this final 5-min test session, the times spent exploring odors in the new and familiar locations were compared.

*Four odor What*: This paradigm was run identically to the Where task (above) except during the test session, rather than changing odor locations, one of the four odors was replaced with a novel odor. Time spent exploring the novel odor versus the average of the three familiar odors was assessed.

*When*: This paradigm was largely identical to the What task, except that an additional odor pair D:D was added to the last step in the training sequence (see Fig. 1c). After exposure to odor pair D:D, the final test session presented two different odors selected from the prior sequence. Untreated mice explored the odor sampled least recently (e.g., odor B more than C). To assess how sequential learning was affected by interval time, additional experiments were run with the interval between odor pair exposures reduced to 30 s and the mice were not removed from the chamber.

*Two-odor discrimination*: The mice were first exposed to odor pair A:A, moved to a holding chamber for 21 min (the same period covered by presentation of odors B and C in the What task) and then returned to the odor chamber and tested for discrimination of a novel odor D from familiar odor A (see Fig. 1f).

All testing was counterbalanced by location of odors and treatment where applicable. Training and testing sessions were video recorded and later analyzed by an individual blind to group and treatment. A mouse was scored as exploring an odor when its nose was within 2 cm of, and directed toward, the odor hole. Odorants used in the above tests were as follows: (+)-Limonene (≥97% purity, Sigma-Aldrich); cyclohexyl ethyl acetate (≥97%, International Flavors & Fragrances Inc.); (+)-Citronellal (~96%, Alfa Aesar); octyl aldehyde (~99%, Acros Organics); Anisole (~99%, Acros Organics); 1-pentanol (~99%, Acros Organics).

Automated software (Ethovision XT, Noldus) was used to analyze locomotor activity (distance traveled) during the spatial tests.

### Regional DREADD inhibition with behavioral testing

*Stereotaxic surgery:* Mice were anesthetized with ketamine and xylazine (100 and 10 mg/kg IP, respectively), and DREADD construct AAV8-CaMKIIa:HA-hM4Di-IRES-mCitrine (University of North Carolina Vector Core) or AAV8: pAAV-CaMKIIa-hM4D(Gi)-mCherry (Addgene, Watertown MA) was infused (2–3 min) into the target region using a 10-µl syringe with a 33GA metal needle (Hamilton, Reno, NV) as described^36^. The construct was infused bilaterally into LEC or MEC, or unilaterally into CA3b or CA1 using the following coordinates (AP, ML, and DV mm from suture) and quantities: for LEC (from lambda) +1.0, ±4.0, −4.8 (0.8 µl); for MEC (from lambda)+0.13, ±3.3, −4.3 (0.8 µl); for CA3 (from bregma) −1.8, ±3.0, −2.4 (0.3 µl) and −2.9, ±2.87, −4.3 (0.5 µl) and for CA1 (from bregma) −1.85, ±1.85, −1.7 (0.3 µl) and −2.9, ±3.87, −3.65 (0.5 µl). After surgery, mice were returned to their home cage for ≥4 weeks before behavioral testing to allow for virus expression.

*Behavioral tests*: Mice from within a housing group were randomly assigned to receive an intraperitoneal injection of CNO (5 mg/kg; Tocris or National Institute of Mental Health) or vehicle (1% DMSO in saline) 30 min before the onset of cue sampling. Animals with Gi-DREADD injections in each of the three target regions (LEC, MEC, unilateral CA3) were tested on What, Where, or When tasks. To avoid possible carry over effects between tasks, separate cohorts were used for each behavior; there were thus three CNO groups for each transfection site. We found no differences between the effects of vehicle injections in the transfected mice from those recorded for untreated mice and accordingly combined their data for each of the three behavioral tests.

*Histology*: Animals were anesthetized after the conclusion of behavioral testing and decapitated; the forebrain was cryostat sectioned at 30 µm. Placement of the AAV-DREADD injections was verified by fluorescence microscopy to localize transfected neurons and fluorescent labeling of known projections from the target sites to specific hippocampal subfields throughout the septo-temporal extent of the structure (LEC to outer molecular layer of the dentate gyrus (DG); MEC to middle molecular layer of the DG; ipsilateral CA3 gives rise to bilateral projections to apical and basal dendrites of CA1-CA3). Three to four 370 µm thick hippocampal slices were prepared from a subgroup of animals and used for electrophysiological analyses of the extent to which CNO depressed synaptic transmission; these slices were then fixed in 4% paraformaldehyde, and subsectioned parallel to the broad slice face (30 µm). These subsections were slide mounted and processed for immunohistochemistry to enhance localization of the construct-associated fluorescent marker. Primary antisera used were either chicken anti-GFP (ab13970, abcam) or rat anti-mCherry (M11217, Invitrogen) both at 1:1000 dilution. Secondary antisera used were Alexa Fluor 488 goat anti-chicken IgG (A11039, Life Technologies) and Alexa Fluor 594 donkey anti-rat IgG (A21209, Invitrogen), both at 1:1000 dilution. Images were collected using a Leica DMI6000B epifluorescence microscope with an sCMOS pco.edge camera and MetaMorph v7.8.7.0 software. Animals that did not satisfy criteria of correct injection placement and fluorescent labeling of the appropriate projection system were not included in the analysis of behavioral or electrophysiological data.

### Hippocampal slice electrophysiology

*Evoked responses in perforant path:* Transverse hippocampal slices (370 µm thick) were prepared using a Leica VT1000 vibroslicer, and transferred to an interface recording chamber with constant oxygenated aCSF perfusion (60–70 ml/h; 31 ± 1 °C) as described^36,38^. The extracellular aCSF solution (ECS) contained (in mM): 124 NaCl, 26 NaHCO_3_, 3 KCl, 1.25 KH_2_PO_4_, 2.5 CaCl_2_, 1.5 MgSO_4_ and 10 glucose and (pH 7.4, 300–310 mOsm). Recordings began ~1.5 h after slices were transferred to the chamber. Throughout, fEPSPs were elicited using a twisted nichrome wire stimulating electrode and recorded with a glass pipette electrode (2 M NaCl; R = 2–3 MΩ)^36,38^. Single pulse baseline stimulation was applied at 0.05 Hz with intensity adjusted to achieve 50–60% of the maximum spike-free fEPSP. All recordings were digitized at 20 kHz using an AC amplifier (A–M Systems, Model 1700) and sweeps of 1.5 s duration were recorded every 20 s using NAC 2.0 Neurodata Acquisition System (Theta Burst Corp. Irvine, CA).

For DREADD experiments, responses were elicited and recorded from the internal blade of the dentate gyrus middle molecular layer with the accuracy of electrode placement verified using paired-pulse stimulation (LPP responses show paired-pulse facilitation and MPP responses show paired-pulse depression^71^). Stable baseline responses were collected for at least 20 min prior to the infusion of CNO (10 µM) via a second infusion line.

*Recordings of pyramidal cell spikes from CA3*: Slices were prepared from the temporal hippocampus as previously described^39,72^. Brains were rapidly removed and placed in ice cold, oxygenated (95% O_2_), high magnesium aCSF containing (in mM): 87 NaCl, 75 sucrose, 26 NaHCO_3_, 2.5 KCl, 1.25 NaH_2_PO_4_, 0.5 CaCl_2_, 7 MgCl_2_ and 10 glucose (pH 7.4, 320–335 mOsm). Horizontal slices (400 µM) were cut using a Leica VT1000 at 0–4 °C and placed on an interface recording chamber (31 ± 1 °C; 95% O_2_/5% CO_2_). Oxygenated ECS (as above) was perfused at a rate of 60-70 ml/h and recordings began ~1.5 h after transfer to the chamber.

Glass pipettes (*R* = 2–3 MΩ) containing 2 M NaCl were placed in CA3a/b stratum pyramidale to record PC spiking and evoked fEPSPs. Tests for evoked PC spiking used stimulating electrodes placed in CA3b/c and CA2/CA1 stratum radiatum (see Fig. 3a). The current intensity was adjusted so that dual stimulation produced a small composite fEPSP that typically was not accompanied by an antidromic spike. PC spiking was recorded in 10 s sweeps, separated by 10 s intervals (i.e., 3 sweeps per minute). Baseline measures were established for 2.5 min (i.e., seven, 10 s epochs) prior to assessing the effect that a 10-pulse Θ train (5 Hz) had upon the frequency and duration of PC spiking. This stimulation protocol was repeated 2–3 times per slice, with 10 min between trains. Spiking was analyzed offline using custom-written computer code created with Python (v3.5), NumPy, and SciPy. Extracellular recordings were fed through a Butterworth band pass filter (300–3000 Hz). There was considerable slice-to-slice variability in the amplitude of spikes and counting thresholds were adjusted accordingly (100.1 ± 5.5 µV, mean ± SEM). The number of PC spikes was measured in 1 s bins and an average value was calculated for each 10 s epoch. A mean frequency was measured over the 140 s baseline period (i.e., seven 10 s epochs spaced by 10 s) after which a 10-pulse Θ train was delivered and spike frequency similarly calculated for an additional 4 min. Trials were dropped from the analysis if the coefficient of variation (CV) for the seven baseline epochs was greater than 45%; this occurred in 10.6% of the experiments. Mean baseline and post-stimulation PC spike frequencies were calculated for all (2–3) trials for a given slice. For quantitative comparison between trials, within and across slices, the post-Θ data were normalized to their respective baseline frequency.

A separate set of experiments compared the effects of partially inhibiting the CA3 associational system, either through activation of G_i_-DREADDs or by reducing ionotropic glutamate receptor function with 500 µM kynurenic acid (KYN, Sigma Aldrich), on spontaneous PC spiking and the amplitude of synaptic responses. A recording pipette was positioned in the CA3b PC layer to record spikes and the positive end of the fEPSP dipole. A single stimulating electrode was placed in CA1 stratum radiatum to activate Schaffer-commissural fibers terminating in the proximal apical dendrites of CA3. Single pulses elicited a fast antidromic population spike superimposed on the rising phase of the synaptic response (see Fig. 4f). Pulses were applied at 3/min with a current intensity that produced stable fEPSP amplitudes throughout a 20-min period of baseline recording. Responses were collected for at least 20 min prior to the application of KYN (40 min), CNO (10 µM, 30–40 min), or vehicle (30–40 min) to slices prepared from naive or G_i_-DREADD expressing mice. Recordings were digitized at 20 kHz and sweeps of 10 s duration recorded every 20 s (i.e., 10 s between sweeps) using NAC 2.0 Neurodata Acquisition System (Theta Burst Corp. Irvine, CA). The maximum amplitude of the fEPSP was measured during the 10 ms following the stimulus artifact.

*Recordings of SPWs*: Although electrode placement was optimized to record PC spiking from field CA3, it was also possible, in a number of cases, to detect SPWs. The manner in which Θ train stimulation influenced the frequency of SPWs and the properties of the waveform were assessed in cases where prolonged PC spiking was observed. SPWs were analyzed offline using the Strathclyde Electrophysiological Software (Electrophysiological Data Recorder [Win EDR] and Whole Cell Analysis Program [Win WCP] courtesy of Dr John Dempster, University of Strathclyde). Using WinEDR, SPWs were detected using an amplitude threshold detection algorithm (50 μV threshold). The SPW frequency was quantified for each 10 s sweep during the baseline period (2.5 min) and following Θ stimulation (3 min). Detected events were visually inspected to ensure any spurious noise was removed. Trials where the baseline frequency was less than 0.5 Hz were not included in the analysis (~50% of all cases with prolonged firing).

In order to analyze basic properties, individual SPWs were visually inspected and examples containing multiple events (i.e., complex SPW) or electrical noise were rejected. Accepted events, typically 10–20 for each experimental condition (i.e., prestimulation and poststimulation) were digitally averaged. Individual SPWs were analyzed with regard to peak amplitude, rise time and decay kinetics (T50% and T90% values; the time taken for the amplitude of each event to decay to 50 and 90% of peak, respectively). The decay phase of the digitally averaged SPW was fitted with a single exponential [Y(*t*) = A*exp(-*t*/τ)] to produce a decay τ for each.

*Whole-cell current clamp recordings*: Hippocampal slices were prepared as described (above) and then incubated in a recovery chamber for ~1 h in oxygenated high magnesium aCSF, before being transferred to a holding chamber and incubated at room temperature (20–22 °C) in ECS. Slices were then transferred to the recording chamber as required.

Current clamp recordings from CA3 and CA1 PCs were made at 30–32 °C in ECS, using patch pipettes (*R* = 5–8 MΩ) filled with an intracellular solution containing (in mM): 135 K-gluconate, 10 HEPES, 4 KCl. 1 MgCl_2_, 2 Mg-ATP, 0.3 Na-GTP, and 10 *Tris*-phosphocreatine (pH 7.2–7.3, 300–310 mOsm). Mean membrane potential was measured offline using WinEDR (Strathclyde Electrophysiology Software, Dr J. Dempster, University of Strathclyde, UK). Specifically, the membrane potential was sampled every 12.8 ms over a 10 s period. At a sampling rate of 10 kHz, 128 baseline points for each 12.8 ms provided one data point. Epochs containing action potentials were excluded. The maximum change in membrane potential (Δ*V*_memb_) was calculated as: (Max.*V*_memb_ − Min.*V*_memb_).

*Drug application*: For hippocampal slice experiments, compounds were introduced to the bath via a second, independent perfusion line (6 ml/h). CNO was made as a concentrated stock (1000×) in DMSO and then diluted to the bath concentration (10 µM) in ECS. The maximum DMSO concentration (≤0.01%) had no effect on baseline transmission. Kynurenic acid was dissolved directly into ECS to achieve a final bath concentration of 500 µM.

### Simulation of a CA3 Neuronal Network

We modified a previously described DG-CA3-CA1 model^39^ to test the hypothesis that field CA3 is capable of generating prolonged, reverberating activity and to investigate features necessary to produce such simulated activity. CA3 was comprised of 1000 PCs and 100 interneurons providing a 10:1 ratio of excitatory to inhibitory neurons. PCs and interneurons were modeled by the two-compartment Pinsky-Rinzel model^73,74^ and the single compartment Wang–Buzsaki model^75^ respectively. The PCs were recurrently connected to each other, as well as to inhibitory interneurons resulting in strong feedforward and feedback inhibition. Each PC had a substantially high probability of connection to its 25 neighbors than to more distant ones. Mossy fiber terminals released stochastically onto each PC. Excitatory and inhibitory synaptic connections between cells were mediated by AMPA and GABA_A_ receptors, respectively. Synaptic interactions were modeled as previously described^39,76^. Briefly, currents mediated by AMPA and GABA_A_ receptors are described by:

$$I_{\rm{syn}} = \bar g_{\rm{syn}}s_{\rm{syn}}\left( {V - V_{\rm{syn}}} \right)$$

where *ḡ*_syn_ is the maximum conductance, *V* is the membrane potential of the postsynaptic neuron, *V*_syn_ is the reversal potential of the synapse, and *s*_syn_ is a gating variable that decreases exponentially:

$$\frac{{ds_{\rm{syn}}}}{{dt}} = - \frac{{s_{\rm{syn}}}}{{\tau _{\rm{syn}}}}$$

where *τ*_syn_ is the decay time constant. In the postsynaptic neuron, *s*_syn_ is increased by a fixed value, *α*_syn_, following the arrival of an action potential at the synapse. Conductance for different types of synapses, and thus the magnitude of the postsynaptic potentials they produce, is controlled by the variable *α*_syn_. The values for *α*_syn_, scaled from a 1 nS base, were set as follows: (PC-PC:17; PC-interneuron: 5; interneuron–PC: 35; interneuron-interneuron: 35). The probability that an interneuron formed a connection was 30% of that for the PCs. The decay time (*τ*) of excitatory currents was 4 ms for CA3–CA3 synapses while those for inhibitory currents on PCs and interneurons were 6 and 2 ms, respectively. Response facilitation occurred at PC–PC contacts when active inputs caused the target cell to spike and were followed within 20 ms by a second excitatory input; initial potentiation was 2× of the baseline and decayed at 0.001/ms. Variation of the reversal potential was distributed across neurons using a Gaussian distribution in order to introduce heterogeneity to the system. A firing reset voltage of −60 mV was introduced. A small collection of weak excitatory synapses (α_syn_: 3) firing at rates set by a Poisson distribution (mean 10 Hz) was included to add noise to the system.

The network was activated using 10 brief 2-ms trains of input delivered at 10 Hz to small collections of PCs scattered throughout the system. Spiking in response to the input was measured from a group of four neighboring neurons to mimic physiological recording from a single electrode. The effects of fluctuations in membrane potentials across the CA3 network on evoked activity were tested by placing the model in one of two states at the time of stimulation. Either a 30% hyperpolarized state or a 70% hyperpolarized state was induced by delivering hyperpolarizing current injections (0.25–1 nA) to randomly selected subpopulations of PCs (30% or 70% of total); in each case current was delivered in two 50-ms epochs to different cohorts of randomly selected neurons (30% or 70%). For the remainder of the run, every 50 ms, the hyperpolarization shifted to new randomly selected PCs, encompassing 70% of the full population. Across each 50-ms window, individual cells linearly ramped to their new target polarization state. Simulated activation of firing was run at least 75 times with the network under the ‘30%’ and ‘70%’ initial states. The duration of prolonged firing was assessed for the two initial states and normalized (for each current amplitude) to 5 s, whereas the probability of prolonged firing was assigned “1” if it exceeded a duration of 500 ms and “0” if it did not.

Tests for the sensitivity of recurrent activity to collapse were made by reducing the conductance of excitatory synapses between PCs only. The effects of changing excitatory conductance upon the duration of firing (normalized to the maximum firing duration) were quantified across a minimum of 50 runs per condition.

All simulations were written in Python 3.5 using the Brian2 spiking neural network simulator with NumPy, SciPy, and matplotlib. Large batches of simulations were run on the University of California at Irvine High Performance Computing Cluster using Unix and Sun Grid Engine.

### Statistics and reproducibility

All results are presented as means ± SEM. Paired Student’s *t* -tests (two-tails) were used to evaluate within group differences in sampling of predicted versus nonpredicted odors with significance set at *p* < 0.05. Differences between groups in the % time sampling predicted odors were evaluated with unpaired *t*-tests (two-tails). In physiological experiments, statistical comparisons between groups (e.g., prestimulation versus poststimulation, positive versus negative cases) were made using Student’s *t* test (paired and unpaired). When normalized data are presented, repeated measures analysis of variance (RM-ANOVA) was used to test for group effects between multiple 10 s sampling bins. All analyses were performed with Prism software (GraphPad; San Diego, CA), which provides evaluation of the suitability of the test for the specific data set. Full statistical values are described in Supplemental Tables 1 and 2.

As noted above, in an effort to reduce the possibility of experimental bias and to enhance reproducibility, animal behavior (cue exploration) was scored from videotapes by individuals blind to treatment and group and the animal’s distance traveled was evaluated using automated systems (Ethovision) and was thus not subject to experimenter bias. Moreover, during testing the positions of the novel and familiar cues were switched (sometimes on the right, sometimes on the left); all odors used in the tasks had been previously evaluated to determine they were of comparable interest to mice. For the DREADD experiments, animals were randomly selected to receive CNO or vehicle prior to behavioral testing and were run together in the same sessions. For electrophysiological studies, baseline PC spiking was recorded for at least 10 min prior to stimulation, and cases where the CV for the seven epochs (2.5 min) immediately before stimulation exceeded 45 were dropped from the analysis. Following bandpass filtering (300–3000 Hz), PC spikes were detected using an amplitude threshold which was determined in a case by case manner to focus upon larger amplitude spikes (i.e., closer to the recording electrode). This approach enabled changes in the activity of PCs proximal to the recording electrode to be detected. When evaluating DREADD efficacy, the experimenter was blind to treatment, and the slices receiving CNO versus vehicle were from the same animals and were run on adjacent slice chambers at the same time.

### Reporting summary

Further information on research design and experimental design is available in the Nature Research Reporting Summary linked to this article.

## Supplementary information

Supplementary Information

Reporting Summary

## Data Availability

The supporting data in this study are available on request from the corresponding authors.

## Code availability

Relevant modelling and analysis code can be found at: https://github.com/cdcox/CoxCoxGunnetal2019
