# Supplementary material for: Acquisition of temporal order requires an intact CA3 commissural/associational (C/A) feedback system in mice
Source: Commun Biol. 2019 Jul 3;2:251. doi: 10.1038/s42003-019-0494-3 (PMC6610080; doi:10.1038/s42003-019-0494-3)
Supplement: Supplementary file 1 — Supplementary Information [file 42003_2019_494_MOESM1_ESM.pdf]

Supplementary Figures

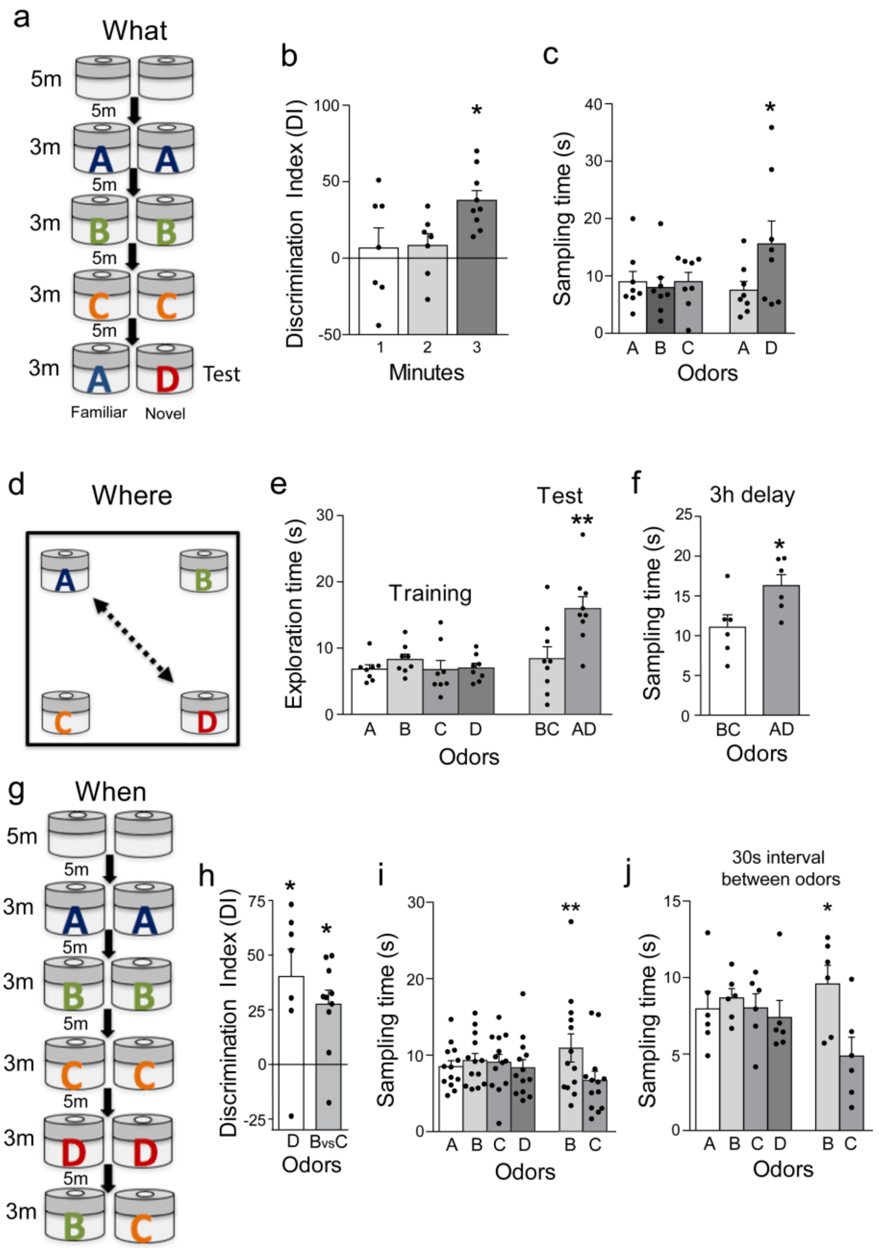

Supplemental Figure 1. Variations in tests for What, Where, and When acquisition. **a** Design of the What retention task using a set of three serial odors (see Fig 1a for details). **b** Effect of initial odor sampling on discrimination in the test trial; for the What task, 3 min sampling was needed for later discrimination of the novel cue D (*$t_{(7)}$=3.17, p=0.016), but not for 1 or 2 min (p>0.05), (Discrimination index: ([novel – familiar] / [novel + familiar])*100). **c** Mean time sampling odors during each phase of the 'what' task; time spent with odor pairs did not differ for odors A, B, C (p>0.05), but mice preferentially explored odor D vs A in the test trial (*$t_{(7)}$=3.17, p=0.016). **d** Design of the spatial/Where test. **e** There was no difference in the time spent with the 4 odors during the initial exposure (p>0.05), but mice preferentially explored odors in novel (AD) vs familiar locations (BC) during testing (**$t_{(8)}$=4.27, p=0.003). **f** Mice retained their preference for odors in the novel locations after a 3 hr delay (BC vs AD *$t_{(5)}$=3.995, p=0.01). **g** Design of the test for acquisition of the order in which odors had been sampled (When). **h** Separate groups of mice were tested on different odor pairings from the When task. During a retention trial, mice spent more time investigating the earlier odor in a previously sampled sequence when the odors were widely separated (A:D) or occurred sequentially (B:C) in the sequence (A vs D:*$t_{(8)}$=3.21, p=0.01, B vs. C: $t_{(8)}$=3.28, p=0.01). **i** Mean time spent sampling the odors in the When test did not differ by position in the sequence (p>0.05), but mice preferentially explored odor B vs C in the test trial (**$t_{(12)}$=3.89, p=0.002). **j** We tested if When learning also occurs with short 30 s intervals between odor pair presentations: as shown the average time spent exploring the odor pairs did not differ across the sampling series (p>0.05), and mice preferentially explored odor B vs C in the test trial (*$t_{(5)}$=3.87, p=0.01).

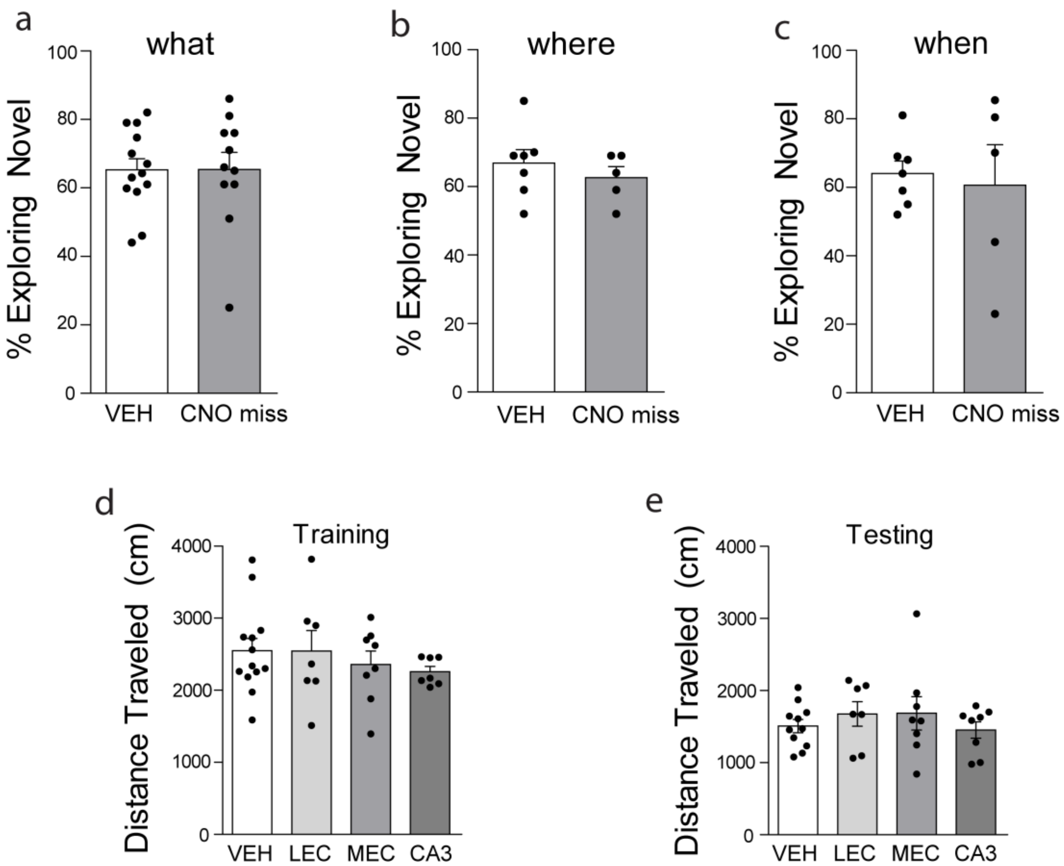

Supplemental Figure 2. CNO effects with and without Gi-DREADD transfection. a-c CNO did not affect acquisition in the What, Where, or When paradigms when DREADD injections missed their targets. Performance of mice that received CNO (CNO miss) did not differ compared to vehicle controls (VEH). Graphs show percent sampling time of the novel odor in the VEH vs. CNO (miss) groups during retention testing for What: $t_{(22)}=0.013$, $p=0.99$; Where: $t_{(10)}=0.79$, $p=0.45$; and When: $t_{(10)}=0.315$, $p=0.75$. **d-e** CNO in mice with appropriately targeted Gi-DREADD transfections did not affect exploratory behavior during initial odor sampling or retention testing. Distance traveled was measured from overhead videos and automated software in the Where chamber, which was large enough to assess exploration. CNO treatment had no effect on distance traveled in any of the three transfection groups (LEC, MEC, CA3) relative to vehicle controls (VEH) during the initial sampling or testing phases (one-way ANOVA, training: $F_{(3,31)}=0.53$, $p=0.66$; testing: $F_{(3,30)}=0.58$, $p=0.63$).

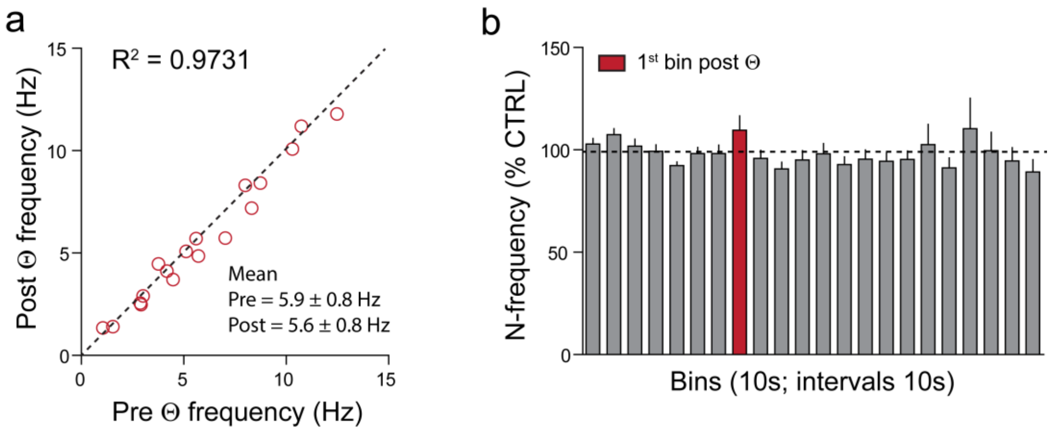

Supplemental Figure 3. A short theta train did not elicit prolonged CA3 firing. a Scatter plot with unity line (dashed) illustrates the mean frequency before and 2 minutes after Θ stimulation for 29 negative trials from 18 slices (p >0.05, paired t-test pre- vs. post stimulation). These values were highly correlated, indicating that mean firing rate was stable across the duration of the experiments. **b** Bar graph summarizing the mean normalized frequency (% baseline) for each 10 s bin (10 s intervals between bins) during the baseline period and for four min following Θ stimulation. There was no evidence for group (bins) differences across the time course of the recording sessions ($F_{(21,357)}$=1.164, p=0.2805).

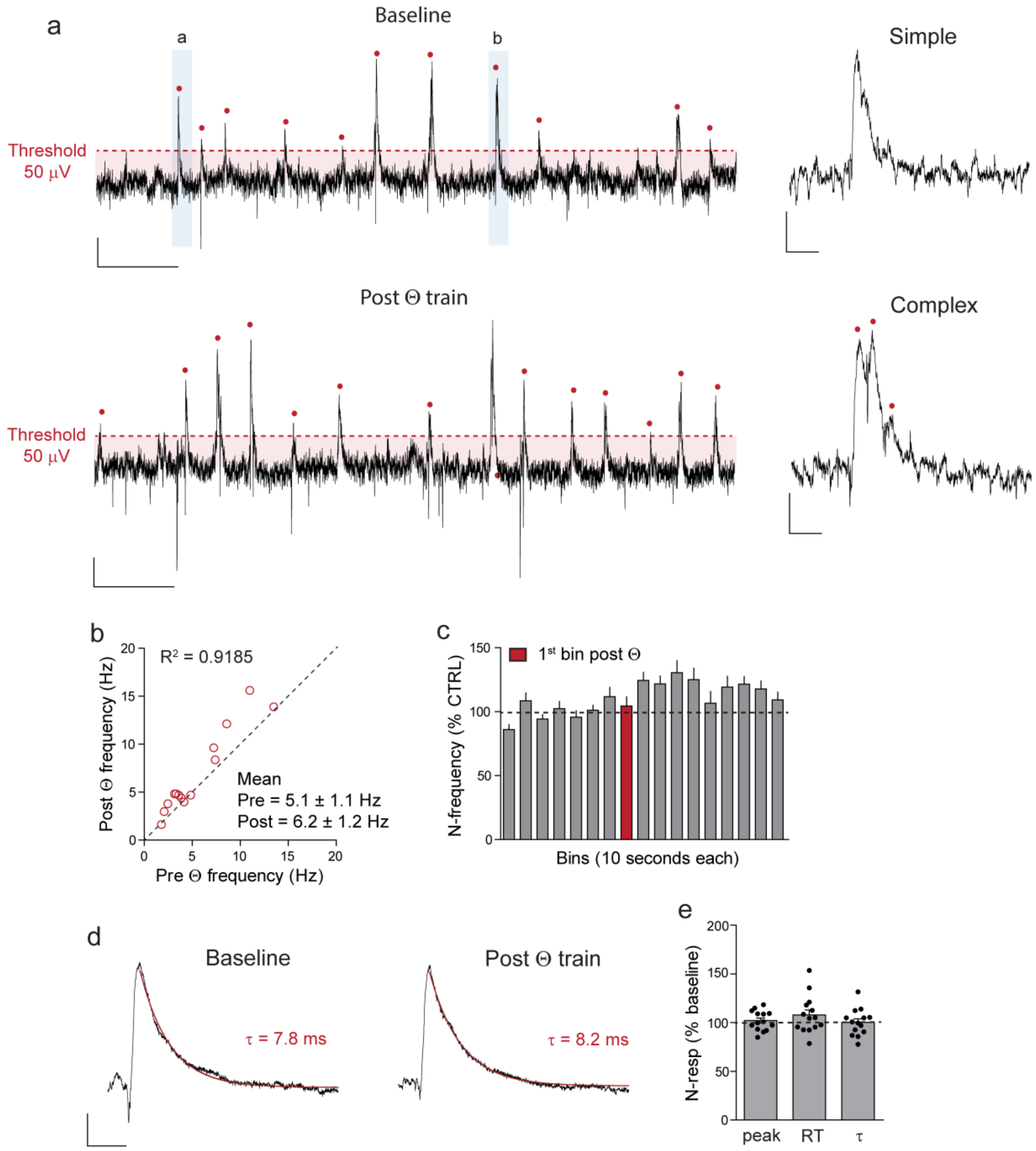

Supplemental Figure 4. CA3 C/A activation increases sharp wave (SPW) frequency. a Representative sections (2s) of extracellular recordings of SPWs during the baseline period and following Θ train stimulation. The detection threshold (50μV) and those events counted during both recording periods are indicated with red shading and red dots respectively. Exemplar simple (blue shaded area 'a') and complex SPWs (blue shaded area 'b') are illustrated on an expanded time scale (right). The complex case has multiple large defections (red dots) riding on the positive going wave. Note that an increase in the incidence of SPWs and PC spiking is evident following theta stimulation. Scale bars: y = 50 μV, x = 250 ms and 10 ms. **b** Scatter plot with unity line (dashed) illustrates the mean SPW frequency before and 2 minutes after Θ stimulation for all prolonged PC spiking cases meeting SPW criteria (~ 50% of cases, see Methods; p <0.01 paired t-test pre- vs. post stimulation). **c** Bar graph summarizing the mean normalized frequency (% baseline) for each 10 s bin (10 s intervals between bins) during the baseline period and for three min following Θ stimulation ($F_{(16,208)}$=4.172, p<0.0001). **d** Normalized ensemble average of simple SPWs recorded before (left) and after (right) Θ stimulation. The decay time course (τ), described by a mono-exponential fit is illustrated for each (red line). **e** Bar graph summarizing the effects of Θ stimulation upon the amplitude, rising phase and decay time course of simple SPWs (p >0.05 paired t test pre- vs post stimulation).

Supplementary Tables

Supplemental Table 1. Statistical values for behavioral analyses.

NO TREATMENT

Fig.		within group comparisons	
**1a**.	WHAT	(odors A vs D)	$t_7 = 3.169$, p=0.0157
**1b**.	WHERE	(odors B-C vs A-D)	$t_8 = 4.266$, p=0.0027
**1c**.	WHEN	(odors B vs C; 5min)	$t_{12} = 3.889$, p=0.0022
		(odors B vs C; 30s)	$t_5 = 3.868$, p=0.0118

red: preferred odor(s)
n=36 mice

Gi-DREADD (VEH & CNO)

	Fig.		within group comparisons	between group comparisons (VEH vs. CNO)
			(odors A vs D)	
WHAT n=62		VEH:	$t_{20} = 3.892$, p=0.0009	
	**1g**.	CNO: LEC	$t_4 = 0.416$, p=0.6984	$t_{24} = 2.518$, p=0.0189
	**2c.**	CNO: MEC	$t_9 = 2.924$, p=0.0192	$t_{28} = 0.339$, p=0.7364
	**5h**.	CNO: CA3	$t_6 = 5.428$, p=0.0016	$t_{26} = 0.043$, p=0.9653
		VEH:	$t_8 = 2.608$, p=0.0312	$t_{17} = 2.817$, p=0.0119
	* **1i**.	CNO: LEC/DG	$t_9 = 0.137$, p=0.894	
			(odors B-C vs A-D)	
WHERE n=36		VEH:	$t_{14} = 3.766$, p=0.0021	
	**1g**.	CNO: LEC	$t_6 = 0.528$, p=0.6162	$t_{15} = 3.293$, p=0.004
	**2c.**	CNO: MEC	$t_7 = 1.571$, p=0.1601	$t_{21} = 2.405$, p=0.0255
	**5h**.	CNO: CA3	$t_5 = 3.430$, p=0.0186	$t_{19} = 0.039$, p=0.1193
			(odors B vs C)	
WHEN n=53		VEH:	$t_{13} = 3.823$, p=0.0021	
	**1g**.	CNO: LEC	$t_6 = 1.156$, p=0.2918	$t_{19} = 2.215$, p=0.0392
	**2c.**	CNO: MEC	$t_{14} = 2.201$, p=0.0445	$t_{20}= 0.032$, p=0.9742
	**5h**.	CNO: CA3	$t_7 = 1.115$, p=0.3017	$t_{20} = 4.055$, p=0.0006
	**5i**.	CNO: CA1	$t_8 = 2.978$, p=0.0177	$t_{21} = 0.023$, p=0.9817
			(odors A vs D)	
2-ODOR TESTs n=23		VEH:	$t_4 = 3.575$, p=0.0233	$t_8 = 1.216$, p=0.2585
	**1f**.	CNO: LEC	$t_4 = 5.592$, p=0.0050	
		VEH:	$t_5 = 3.281$, p=0.0220	$t_{11} = 0.652$, p=0.5275
	**2d.**	CNO: MEC	$t_6 = 3.071$, p=0.022	
			(odors A-B-C vs E)	
4-ODOR WHAT n=16		VEH:	$t_7 = 3.22$, p=0.0146	$t_{15} = 0.26$, p=0.7984
	**2f.**	CNO: MEC	$t_8 = 5.5$, p=0.0006	

within group: paired t-tests (2-tails) between groups: unpaired t-tests (2-tails)

* contralateral disconnection

Supplemental Table 2. Statistical values for spiking frequencies.

Fig.				
	**Reverberations: All Experiments**			
**3c.**	linear regression (pre-vs. post stim.)	$F_{(1,21)} = 73.74$	$r^2 = 0.778$	$p<0.0001$
**3c.**	mean firing rate (pre-vs. post stim.)	$t_{22} = 3.322$		$p=0.0031$
**3d.**	RM-ANOVA (pre-& post stim.)	$F_{(21,462)} = 3.378$		$p<0.0001$
	**Reverberations: Positive Cases**			
**3e.**	linear regression (pre-vs. post stim.)	$F_{(1,17)} = 56.18$	$r^2 = 0.768$	$p<0.0001$
**3e.**	mean firing rate (pre-vs. post stim.)	$t_{18} = 3.322$		$p<0.0001$
**3f.**	RM-ANOVA (pre-& post stim.)	$F_{(21,378)} = 5.483$		$p<0.0001$
	**Reverberations: Negative Cases**			
**S3a.**	linear regression (pre-vs. post stim.)	$F_{(1,16)} = 579.2$	$r^2 = 0.973$	$p<0.0001$
**S3a.**	mean firing rate (pre-vs. post stim.)	$t_{17} = 1.983$		$p=0.0638$
**S3b.**	RM-ANOVA (pre-& post stim.)	$F_{(21,357)} = 1.164$		$p<0.2805$
	**Sharp wave frequency: All Experiments**			
**S4b.**	linear regression (pre-vs. post stim.)	$F_{(1,12)} = 135.3$	$r^2 = 0.919$	$p<0.0001$
**S4b.**	mean firing rate (pre-vs. post stim.)	$t_{13} = 3.503$		$p=0.0039$
**S4c.**	RM-ANOVA (pre-& post stim.)	$F_{(16,208)} = 4.172$		$p<0.0001$
	**fEPSP vs spiking**			
**4g.**	mean fEPSP ampl.	$t_3 = 6.052$		$p=0.0091$
	mean firing rate *(baseline vs KYN)*	$t_3 = 23.50$		$p=0.0002$
	fEPSP vs spiking	$t_3 = 13.93$		$p=0.0008$
**5g.**	mean fEPSP ampl.	$t_2 = 2.628$		$p=0.1194$
	mean firing rate *(CTRL: baseline vs CNO)*	$t_4 = 3.720$		$p=0.6107$
	mean fEPSP ampl.	$t_7 = 2.757$		$p=0.0282$
	mean firing rate *(DREADD: baseline vs CNO)*	$t_5 = 3.983$		$p=0.0105$
	mean fEPSP ampl.	$t_9 = 2.261$		$p=0.0502$
	mean firing rate *(CTRL vs DREADD)*	$t_8 = 8.644$		$p<0.0001$

Supplemental Table 3

	Baseline	Post Θ train	Statistics *(paired Student's test)*
Peak amplitude (μV)	87.0 ± 5	88.4 ± 5	$p=0.611$; $t_{13}=0.521$
Rise time (ms)	1.1 ± 0.1	1.1 ± 0.1	$p=0.288$; $t_{13}=1.107$
T50% (ms)	4.5 ± 0.2	4.4 ± 0.3	$p=0.278$; $t_{13}=1.133$
T90% (ms)	7.6 ± 0.6	7.8 ± 0.6	$p=0.951$; $t_{13}=0.062$
τ (ms)	5.3 ± 0.3	5.3 ± 0.3	$p=0.949$; $t_{13}=0.065$
Frequency (Hz)	5.5 ± 1.0	6.5 ± 1.1	$p=0.0039$; $t_{13}=3.503$

Supplemental Table 3. Summary of SPW properties. (n = 14 trials derived from 10 slices).
