# Supplementary material for: Acquisition of temporal order requires an intact CA3 commissural/associational (C/A) feedback system in mice
Source: Commun Biol. 2019 Jul 3;2:251. doi: 10.1038/s42003-019-0494-3 (PMC6610080; doi:10.1038/s42003-019-0494-3)
Supplement: Supplementary file 2 — Reporting Summary [file 42003_2019_494_MOESM2_ESM.pdf]

nature research

Corresponding author(s): Drs. Gary Lynch & Christine Gall

Last updated by author(s): May 22, 2019

Reporting Summary

Nature Research wishes to improve the reproducibility of the work that we publish. This form provides structure for consistency and transparency in reporting. For further information on Nature Research policies, see Authors & Referees and the Editorial Policy Checklist.

Statistics

For all statistical analyses, confirm that the following items are present in the figure legend, table legend, main text, or Methods section.

n/a	Confirmed	
☐	☒	The exact sample size (n) for each experimental group/condition, given as a discrete number and unit of measurement
☐	☒	A statement on whether measurements were taken from distinct samples or whether the same sample was measured repeatedly
☐	☒	The statistical test(s) used AND whether they are one- or two-sided<br>*Only common tests should be described solely by name; describe more complex techniques in the Methods section.*
☐	☒	A description of all covariates tested
☐	☒	A description of any assumptions or corrections, such as tests of normality and adjustment for multiple comparisons
☐	☒	A full description of the statistical parameters including central tendency (e.g. means) or other basic estimates (e.g. regression coefficient) AND variation (e.g. standard deviation) or associated estimates of uncertainty (e.g. confidence intervals)
☐	☒	For null hypothesis testing, the test statistic (e.g. F, t, r) with confidence intervals, effect sizes, degrees of freedom and P value noted<br>*Give P values as exact values whenever suitable.*
☒	☐	For Bayesian analysis, information on the choice of priors and Markov chain Monte Carlo settings
☒	☐	For hierarchical and complex designs, identification of the appropriate level for tests and full reporting of outcomes
☒	☐	Estimates of effect sizes (e.g. Cohen's d, Pearson's r), indicating how they were calculated

Our web collection on statistics for biologists contains articles on many of the points above.

Software and code

Policy information about availability of computer code

Data collection	We do provide description of commercial data collection software including "NAC 2.0 Neurodata Acquisition System (Theta Burst Corp. Irvine, CA)" and "Automated software (Ethovision XT, Noldus) was used to analyze locomotor activity (distance traveled) during the spatial tests." We further state, "SPWs were analyzed offline using the Strathclyde Electrophysiological Software (Electrophysiological Data Recorder [Win EDR] and Whole Cell Analysis Program [Win WCP] courtesy of Dr John Dempster, University of Strathclyde)."
Data analysis	We state in the manuscript : "Modelling and analysis code can be found at: https://github.com/cdcox/CoxCoxGunnetal2019". This relates to the analysis of electrophysiological recordings and the simulation presented.

For manuscripts utilizing custom algorithms or software that are central to the research but not yet described in published literature, software must be made available to editors/reviewers. We strongly encourage code deposition in a community repository (e.g. GitHub). See the Nature Research guidelines for submitting code & software for further information.

Data

Policy information about availability of data

All manuscripts must include a data availability statement. This statement should provide the following information, where applicable:

- Accession codes, unique identifiers, or web links for publicly available datasets
- A list of figures that have associated raw data
- A description of any restrictions on data availability

The data that supports the findings of this study are avaliable from the corresponding authors (GL, CMG) upon reasonable request.

Field-specific reporting

Please select the one below that is the best fit for your research. If you are not sure, read the appropriate sections before making your selection.

☒ Life sciences ☐ Behavioural & social sciences ☐ Ecological, evolutionary & environmental sciences

For a reference copy of the document with all sections, see nature.com/documents/nr-reporting-summary-flat.pdf

Life sciences study design

All studies must disclose on these points even when the disclosure is negative.

Sample size	Group sizes for the different analyses were selected on the basis of past experience and to be equal to or exceed dictates of power analyses. For electrophysiological studies, power analysis determined that with the typical effect size and profile (e.g., change in response amplitude of 20% ; sigma = 15; alpha = 0.05; power = 0.80) the minimal sample size to determine significance would be 5 slices/group. For behavior experiments similar calculations determined with an effect size (i.e., Discrimination index, DI) of 30% (sigma = 10%; alpha = 0.05; power = 0.80) the minimal sample size to detect a 10% difference in the DI would be 3/group. Because we did not really know the effect sizes to expect we planned on a minimum of 5 per group.
Data exclusions	As described in the manuscript, we verified injection placements into the brain and included for analysis in the bilateral injection group. Only those animals in which injection placement and projections from that injection were verified were included in the experimental group for behavioral studies or used for hippocampal slice analysis of DREADD efficacy. This is explicitly stated in the results section. Behavioral results for animals will 'missed' injections are also described but as a separate group - thus these were not excluded from reporting but rather were placed within another group. We did not exclude results for individual animals, or from hippocampal slices, from analysis or presentation. For electrophysiological analysis, baseline PC spiking was recorded for at least 10 minutes prior to stimulation, and cases where the coefficient of variation (CV) for the 7 epochs (2.5 minutes) immediately before stimulation exceeded 45 were dropped from the analysis.
Replication	All experimental results reported are for groups of animals or slices which constituted independent 'N"s. Hence, the experiment was conducted with each animal or slice and the N represented a replication. We did not conduct full replications with fully separate groups.
Randomization	As stated in the manuscript, for behavioral studies: "Mice from within a housing group were randomly assigned to receive an IP injection of CNO (5 mg/kg; Tocris or National Institute of Mental Health) or vehicle (1% DMSO in saline), 30 min before the onset of cue sampling." For electrophysiological studies with DREADD mice "slices receiving CNO vs vehicle were from the same animals and were run on adjacent slice chambers at the same time". Randomization was not applied for electrophysiological analyses that focused on hippocampal slices from naive mice.
Blinding	As stated all behavioral and studies were rated by observers blind to group. When evaluating DREADD efficacy, the experimenter was blind to treatment.

Reporting for specific materials, systems and methods

We require information from authors about some types of materials, experimental systems and methods used in many studies. Here, indicate whether each material, system or method listed is relevant to your study. If you are not sure if a list item applies to your research, read the appropriate section before selecting a response.

Materials & experimental systems

n/a	Involved in the study	
☐	☒	Antibodies
☒	☐	Eukaryotic cell lines
☒	☐	Palaeontology
☐	☒	Animals and other organisms
☒	☐	Human research participants
☒	☐	Clinical data

Methods

n/a	Involved in the study	
☒	☐	ChIP-seq
☒	☐	Flow cytometry
☒	☐	MRI-based neuroimaging

Antibodies

Antibodies used	chicken anti-GFP (ab13970, abcam), rat anti-mCherry (M11217, Invitrogen), Alexa Fluor 488 goat anti-chicken IgG (A11039, Life Technologies) and Alexa Fluor 594 donkey anti-rat IgG (A21209, Invitrogen)
Validation	We validated both primary antibodies by showing that there was no immunofluorescent labeling in tissue that does not have an AAV viral construct injection (that supports GFP or mcherry associated tag expression); this is similar to a knockout validation.

Animals and other organisms

Policy information about studies involving animals; ARRIVE guidelines recommended for reporting animal research

Laboratory animals	Studies used 2-6 mo old male mice (FVB-129 and C57BL6 backgrounds) group housed (3-5 per cage) with food and water ad libitum. (quoted from manuscript)
Wild animals	none
Field-collected samples	none
Ethics oversight	Experiments were conducted in accord with NIH guidelines for the Care and Use of Laboratory Animals and protocols approved by our Institutional Animal Care and Use Committee.

Note that full information on the approval of the study protocol must also be provided in the manuscript.
